# Distribution of 2,2′,5,5′-Tetrachlorobiphenyl (PCB52) Metabolites in Adolescent Rats after Acute Nose-Only Inhalation Exposure

**DOI:** 10.1021/acs.est.3c09527

**Published:** 2024-03-28

**Authors:** Amanda J. Bullert, Xueshu Li, Binita Gautam, Hui Wang, Andrea Adamcakova-Dodd, Kai Wang, Peter S. Thorne, Hans-Joachim Lehmler

**Affiliations:** † Department of Occupational and Environmental Health, The University of Iowa, Iowa City, Iowa 52242, United States; ‡ Interdisciplinary Graduate Program in Neuroscience, The University of Iowa, Iowa City, Iowa 52242, United States; § Department of Biostatistics, The University of Iowa, Iowa City, Iowa 52242, United States

**Keywords:** acute exposure, disposition, inhalation, metabolites, persistent organic pollutants, PCB52

## Abstract

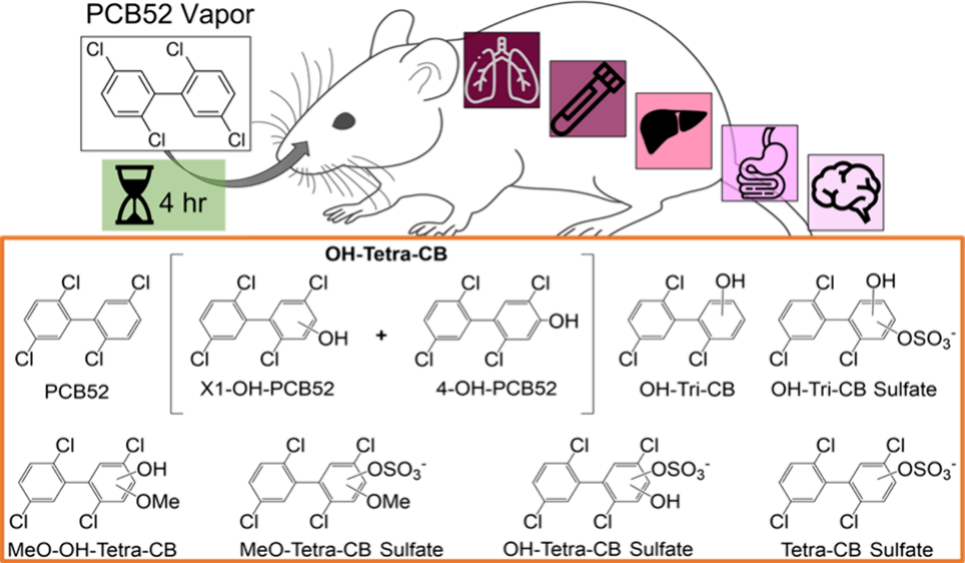

Inhalation of PCB-contaminated air is increasingly recognized as a route for PCB exposure. Because limited information about the disposition of PCBs following inhalation exposure is available, this study investigated the disposition of 2,2′,5,5′-tetrachlorobiphenyl (PCB52) and its metabolites in rats following acute, nose-only inhalation of PCB52. Male and female Sprague–Dawley rats (50–58 days of age, 210 ± 27 g; n = 6) were exposed for 4 h by inhalation to approximately 14 or 23 μg/kg body weight of PCB52 using a nose-only exposure system. Sham animals (n = 6) were exposed to filtered lab air. Based on gas chromatography-tandem mass spectrometry (GC-MS/MS), PCB52 was present in adipose, brain, intestinal content, lung, liver, and serum. 2,2′,5,5′-Tetrachlorobiphenyl-4-ol (4-OH-PCB52) and one unknown monohydroxylated metabolite were detected in these compartments except for the brain. Liquid chromatography-high resolution mass spectrometry (LC-HRMS) analysis identified several metabolites, including sulfated, methoxylated, and dechlorinated PCB52 metabolites. These metabolites were primarily found in the liver (7 metabolites), lung (9 metabolites), and serum (9 metabolites) due to the short exposure time. These results demonstrate for the first time that complex mixtures of sulfated, methoxylated, and dechlorinated PCB52 metabolites are formed in adolescent rats following PCB52 inhalation, laying the groundwork for future animal studies of the adverse effects of inhaled PCB52.

## Abstract 2

Complex mixtures of 2,2′,5,5′-tetrachlorobiphenyl (PCB52) metabolites are formed following acute, nose-only inhalation of PCB52 in adolescent rats.

## Introduction

The United States (US) banned the production of polychlorinated biphenyls (PCBs) in 1979; however, they are still present in building materials and continue to be found in the environment.^1^ They are produced as byproducts in manufacturing consumer products, including paint pigments and polymer resins.^2−4^ One common misconception is that diet^5,6^ is the primary source of current human PCB exposures.^7^ PCBs are present in indoor and outdoor air, with higher PCB air concentrations found inside older school buildings compared to outside,^8,9^ and inhalation is now a major route of PCB exposure in the general population.^10^ These findings raise human health concerns, especially for children who may spend many years in PCB-contaminated indoor environments.^7^ Besides being classified as human carcinogens, PCBs are associated with noncancer end points, including developmental, immune, and reproductive to neurodevelopmental effects.^11^ For example, PCB inhalation is associated with neurotoxic outcomes such as memory impairment,^12^ induced anxiety-like behavior, gene transcription,^13^ and sex differences in response to impulsivity and inhibition tasks in rats.^14,15^

PCBs cause adverse effects, for example, by mechanisms involving the formation of reactive oxygen species, genotoxic effects, immune suppression, inflammatory response, and endocrine disruptive effects.^1,16,17^ Also, the reactive and stable PCB metabolites formed *in vivo* contribute to adverse outcomes.^18^ PCBs and their metabolites have been detected in the rodent and human brain^19^ and are associated with adverse effects on neurological outcomes in children and adolescents.^20−22^ Studies on the harmful effects of PCBs have primarily been based on animal experiments utilizing oral or intraperitoneal (IP) exposure. However, there has been insufficient research on the distribution, metabolism, and toxicity of PCBs following inhalation exposure.^7,8,11,23^ It is challenging for regulatory agencies to assess the human health risks associated with inhaled PCBs because of these knowledge gaps.^23^

Limited laboratory evidence suggests that inhaled PCBs are readily absorbed and distributed via the blood to target tissues, thus bypassing the intestinal and hepatic first-pass metabolism.^24,25^ The disposition of mono- and dichlorinated PCB following inhalation has been reported in a few studies. For example, 3,3′-dichlorobiphenyl (PCB11) and its metabolites are quickly metabolized and eliminated in rats.^24,26^ Intratracheal administration of ^14^C-PCB11 and inhalation of 3-chlorobiphenyl (PCB3) in rats reveal the dominant elimination of more water-soluble metabolites, such as PCB sulfates, with the urine and feces.^24,27,28^ More comprehensive studies of the disposition of individual PCB congeners and their metabolites following inhalation are unavailable for PCB congeners with ≥3 chlorine substituents. Because PCB found in the indoor air of US schools, such as 2,2′,5,5′-tetrachlorobiphenyl (PCB52),^8^ can be metabolized to metabolites that contribute to neurotoxic outcomes,^29−31^ there is a need to characterize the metabolite profiles in target tissue in rodent models following acute, nose-only PCB inhalation, the mode of inhalation exposure preferred by regulatory agencies.^32^

The present study used gas chromatography-tandem mass spectrometry (GC-MS/MS) and liquid chromatography-high resolution mass spectrometry (LC-HRMS) to characterize the metabolite profiles of PCB52 immediately following acute, nose-only inhalation of PCB52 in male and female adolescent rats. PCB52 was selected for this study because it is frequently detected in indoor and outdoor air and human samples and is associated with adverse brain outcomes.^8,10,33^ For the first time, our results demonstrate the presence of complex PCB metabolite mixtures in target tissues, which may affect toxic outcomes following PCB inhalation exposure.

## Materials and Methods

### Chemicals

PCB52 was synthesized by reduction of 2,2′,5,5′-tetrachlorobenzidine with hypophosphorus acid and authenticated as described,^34,35^ and the purity of PCB52 was 99.9% by GC-MS.^35^ 2,2′,5,5′-Tetrachlorobiphenyl-4-ol (4-OH-PCB52) was synthesized by the Suzuki coupling reaction of 2,5-dichloroboronic acid and 2,5-dichloroiodoanisole followed by demethylation.^36^ The PCB surrogate standard, 3,3′,4,4′-tetrachlorobiphenyl (PCB77) was synthesized and authenticated as described.^37^ 2,2′,3,4,4′,5,6,6′-Octachlorobiphenyl (PCB204; internal standard) and 2,3,3′,4,5,5′-hexachlorobiphenyl-4′-ol (4′-OH-PCB159, OH-PCB surrogate standard) were purchased from AccuStandard (New Haven, CT, U.S.A.). For abbreviations and unique identifiers of the test compound and analytical standards, see Table S1.

### PCB52 Vapor Generation

**Figure 1:**
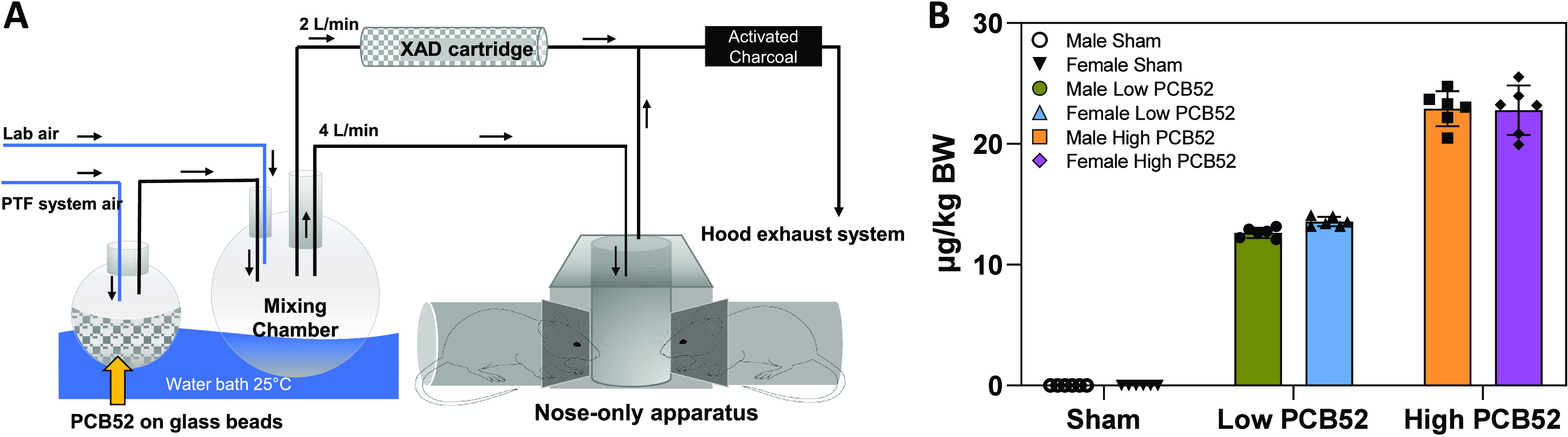
(A) Inhalation apparatus used to expose rats to PCB52 through nose-only inhalation. Briefly, air was passed through glass beads coated with PCB52 at 25 °C in a water bath to volatilize the PCB52. The PCB52-laden air was passed through a mixing chamber and distributed to a nose-only inhalation apparatus at 4 L/min to expose mice to PCB52 vapors and sampling cartridges filled with 10 g of Amberlite XAD-2 polymeric absorbent resin at 2 L/min to capture PCB52 vapor for exposure concentration determination. The arrows indicate the direction of air or PCB vapor flow. (B) Estimated exposure levels in male and female rats exposed to PCB52 vapor for 4 h. Estimates are based on the amount of PCB52 extracted from the XAD powder and estimates of breathing volume, based on body weight, for individual rats. The estimated exposures for male rats were 0.001 ± 0.001, 13 ± 0.4, and 23 ± 1 μg/kg bw for sham, low, and high exposures, respectively. For female rats, the estimated exposures were 0.001 ± 0.001, 14 ± 0.4, and 23 ± 2 μg/kg bw for sham, low, and high exposures, respectively. Bar graphs represent the mean μg/kg bw and standard deviation. Individual points depict individual animals and the variability within groups.

PCB52 vapor for the animal exposure was generated using a published protocol.^12^ Briefly, 450 g of 3 mm diameter glass beads were added to a solution of 1.5 g of PCB52 in dichloromethane. The glass beads were then coated with PCB52 by evaporating dichloromethane in a water bath at 29 °C using a rotary evaporator (Buchi Rotavapor R-200, New Castle, DE) under reduced pressure. For the animal exposures, high-efficiency particulate air (HEPA)-filtered laboratory air was passed through the PCB52-coated glass beads at a flow rate of 12 L/min to expose animals to PCB52 vapor in a nose-only exposure apparatus (Figure 1A). The flask of PCB52-coated beads and mixing chamber were kept at 25.0 °C using a precision water bath to facilitate the volatilization of PCB52. The PCB52-laden air was passed through a mixing chamber and distributed to the nose-only inhalation apparatus at a flow rate of 4 L/min per tower (n = 3) and a sampling cartridge filled with 10 g of Amberlite XAD-2 polymeric absorbent resin (Sigma-Aldrich, St. Louis, MO) at a flow rate of 2 L/min. The specified flow rates were generated using flow meter-regulated pumps. The XAD-2 cartridge was used to capture PCB52 vapor to characterize the exposure atmosphere. A sham exposure group was exposed in parallel to HEPA-filtered clean air in an identical nose-only exposure system in an adjacent lab with no supplied PCB52.

### Animals and PCB52 Exposure

The Institutional Animal Care and Use Committee of the University of Iowa approved all of the animal protocols. Animals were housed in a temperature- and humidity-controlled room (21 °C, 55% relative humidity) with a 12 h light/dark cycle in an Association for the Assessment and Accreditation of Laboratory Animal Care (AAALAC) accredited animal facility. Animals were provided with food (sterile Teklad 5% stock diet, Harlan, Madison, WI) and water *ad libitum* throughout the study. Thirty-six male and female Sprague–Dawley rats (7 weeks of age; Charles River, Wilmington, MA) were allowed to acclimate to the animal facility for 8 days after arrival. Prior to exposure, rats (227 ± 27 and 197 ± 13 g, male and female, respectively) were randomly assigned to the exposure groups (n = 6 per group) using a random number generated (Excel, version 2307). Animals were simultaneously exposed for 4 h to a low or high concentration of PCB52-laden per sex) or filtered lab air (Figure 1A). Exposure duration was limited to 4 h along with scheduled training days (30 min at 55 days of age and 2 h at 56 days of age) to acclimate animals and minimize stress. Sentinel rats (n = 2), housed in the vivarium for health surveillance, showed no evidence of adverse health. Animal exposure occurred for 4 h at 57 days of age. Rats are considered adolescents at this age.^38^ Serum was collected immediately after the animals were removed from the nose-only apparatus by cardiac puncture under isoflurane anesthesia. Rats were perfused with saline, and organs were excised and stored at −80 °C. Body and organ weights, including brain, lungs, and thymus, were not altered by PCB52 inhalation regardless of sex or exposure (Tables S2 and S3).

### Determination of the PCB52 Dose

The PCB52 dose was determined by sampling the exposure atmosphere using an XAD-2 cartridge throughout the inhalation exposure experiments.^12,13^ Briefly, PCB52 in XAD-2 was extracted using pressurized liquid extraction and quantified by GC-MS/MS. The estimated concentrations of PCB52, at a flow rate of 2 mL/min (Figure 1A), were 0.01 ± 0.01, 65 ± 0.3, and 98 ± 30 μg/m^3^ of PCB52 in the sham, low, and high exposure groups, respectively. The dose of PCB52 was determined using the equation:
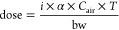
assuming a breath volume *i* m^3^/day based on the animal body weight in kg (*i* = 0.702 × bw^2/3^),^39^ and uptake from the lungs α of 99.89%. *C*_air_ is the experimental PCB exposure concentration (μg/m^3^), and *T* is the duration of exposure (1/6 day).^12^ Based on the variables provided, the estimated exposures for males were 0.001 ± 0.001 μg/kg bw for sham, 13 ± 0.4 μg/kg bw for low exposures, and 23 ± 1 μg/kg bw for high exposures. Similarly, for females, the estimated exposures were 0.001 ± 0.001 μg/kg bw for sham, 14 ± 0.4 μg/kg bw for low, and 23 ± 2 μg/kg bw for high exposures, as shown in Figure 1B.

### Extraction and Quantification of PCB52 and Its Metabolites

PCB52 and its hydroxylated metabolites were extracted from tissues, serum, and cecum and intestinal content using a published protocol;^40,41^ see the Supporting Information. Briefly, PCB52 and its metabolites were extracted from homogenates by liquid–liquid extraction and cleaned by solid-phase extraction with acidified silica gel. PCB52 and metabolites were quantified in the multiple reaction monitoring setting (MRM) on an Agilent 7890 A GC system equipped with an Agilent 7000 Triple Quad and Agilent 7693 autosampler (Agilent Technologies, Santa Clara, CA); see the Supporting Information.

### Extraction and Processing of LC-HRMS Data

Tissues (brain, cecum and intestinal content, liver, lung, and serum) were homogenized and extracted for LC-HRMS analysis following a published approach; see the Supporting Information.^19^ Extracts were analyzed at the High-Resolution Mass Spectrometry Facility at the University of Iowa on a Q-Exactive Orbitrap mass spectrometer (Thermo Fisher Scientific, Waltham, MA, U.S.A.) with an Axquity UPLC-C18 column (particle size: 1.7 μM, 2.1 × 100 mm, Waters, Milford, MA, U.S.A.); see the Supporting Information. Accurate masses and peak areas of PCB52 metabolites were extracted based on a subject screening list of known or likely PCB52 metabolites from raw data files with Thermo Xcalibur (version 4.1, Thermo Fisher Scientific) with a *m*/*z* tolerance of 5 ppm, mass precision decimals of 5, and the smoothing factor of 7 (Table 1).^18,30,31,43^ The isotopic pattern of chlorine was used to confirm the identification of putative metabolites.^29,30,43^

**Table 1:** PCB52 Metabolites Were Detected by LC-HRMS in Tissues from Male and Female Rats Exposed to PCB52 via Nose Only^a^^,^^b^

							**[M–H]**^**–**^			
**Class No.**	**Metabolite**	**Analyte Abr.**	**RT (min)**	**RRT (min)**	**Normalized Intensity, Low Dose**^c^	**Normalized Intensity, High Dose**^c^	**Calculated (Da)**	**Measured (Da)**	**δ**_***m*/*z***_, **δppm**^d^	**Confidence Level**^e^
**Brain**										
1.1.2	OH-Tetra-CB	T2	7.700	1.130	7 ± 3 (*n* = 4)	6 ± 5 (*n* = 7)	304.91000	304.90996	–0.14	3
1.1.4		T4	7.811	1.147	19 ± 3 (*n* = 10)	25 ± 14 (*n* = 9)	304.91000	304.91014	0.46	3
3.1	OH-Tri-CB	Z1	7.692	1.130	8 ± 5 (*n* = 8)	16 ± 9 (*n* = 9)	270.94897	270.94921	0.89	3
**Cecum**										
1.1.2	OH-Tetra-CB	T2	7.715	1.134	5200 ± 5600 (*n* = 12)	11000 ± 16000 (*n* = 8)	314.94687	314.94643	–1.40	3
3.1	OH-Tri-CB	Z1	7.674	1.128	353 (*n* = 1)	443 (*n* = 1)	270.94897	270.94926	1.08	3
**Intestinal content**										
1.1.2	OH-Tetra-CB	T2	7.737	1.138	140 ± 60 (*n* = 12)	80 ± 60 (*n* = 7)	304.91000	304.91024	0.80	3
1.1.4		T4	7.818	1.151	600 ± 400 (*n* = 3)	3000 ± 2900 (*n* = 3)	304.91000	304.91008	0.27	3
3.1	OH-Tri-CB	Z1	7.691	1.131	ND	1500 ± 400 (*n* = 3)	270.94897	270.94879	–0.65	3
**Liver**										
1.1.1	OH-Tetra-CB	T1	6.618	0.976	22 ± 9 (*n* = 3)	ND	304.91000	304.91038	1.25	3
1.1.2		T2	7.689	1.134	15 ± 11 (*n* = 4)	0.02 (*n* = 1)	304.91000	304.91054	1.76	3
1.1.4		T4	7.798	1.150	27 ± 3 (*n* = 3)	ND	304.91000	304.91034	1.12	3
1.2	Tetra-CB Sulfate	U1	6.410	0.945	ND	0.007 (*n* = 1)	384.86681	384.86695	0.37	2
2.1.2	OH-Tetra-CB Sulfate	V2	6.760	0.997	ND	0.006 ± 0.004 (*n* = 7)	400.86173	400.86228	1.36	2
2.2	MeO-OH-Tetra-CB	W1	7.844	1.157	ND	0.007 ± 0.006 (*n* = 5)	334.92056	334.92124	2.02	3
3.1	OH-Tri-CB	Z1	7.673	1.132	20 ± 2 (*n* = 3)	ND	270.94897	270.94955	2.15	3
**Lung**										
1.1.2	OH-Tetra-CB	T2	7.703	1.135	20 ± 20 (*n* = 8)	30 ± 20 (*n* = 10)	304.91000	304.91001	0.03	3
1.1.4		T4	7.813	1.151	4 ± 3 (*n* = 8)	15 ± 19 (*n* = 8)	304.91000	304.91015	0.48	3
1.2	Tetra-CB Sulfate	U5	6.406	0.944	280 ± 160 (*n* = 10)	370 ± 300 (*n* = 12)	384.86681	384.86731	1.31	2
2.1.1	OH-Tetra-CB Sulfate	V1	5.610	0.826	10 ± 6 (*n* = 4)	26 (*n* = 1)	400.86173	400.86180	0.18	2
2.1.2		V2	6.764	0.997	120 ± 60 (*n* = 10)	140 ± 70 (*n* = 12)	400.86173	400.86180	0.18	2
2.3	MeO-Tetra-CB Sulfate	X1	6.216	0.916	15 ± 8 (*n* = 10)	21 ± 10 (*n* = 11)	414.87738	414.87763	0.61	2
3.1	OH-Tri-CB	Z1	7.680	1.133	ND	8 (*n* = 1)	270.94897	270.94931	1.26	3
4.1.1	OH-Tri-CB Sulfate	Y1	5.990	0.882	ND	0.6 ± 0.002 (*n* = 2)	366.90070	366.89852	–5.96	2
4.1.2		Y2	6.280	0.926	ND	3 ± 3 (*n* = 5)	366.90070	366.89759	–8.47	2
**Serum**										
1.1.1	OH-Tetra-CB	T1	6.572	0.963	420 (*n* = 1)	ND	304.91000	304.90963	–1.23	3
1.1.2		T2	7.706	1.130	8 ± 3 (*n* = 6)	20 ± 30 (*n* = 3)	304.91000	304.91002	0.07	3
1.1.3		T3	7.485	1.144	13 ± 3 (*n* = 2)	ND	304.91000	304.90982	–0.59	3
1.1.4		T4	7.815	1.146	580 (*n* = 1)	450 ± 40 (*n* = 2)	304.91000	304.91002	0.07	3
1.2	Tetra-CB Sulfate	U1	6.375	0.948	250 ± 260 (*n* = 12)	330 ± 270 (*n* = 7)	384.86681	384.86582	–2.58	2
2.1.2	OH-Tetra-CB Sulfate	V2	6.702	0.996	1200 ± 1201 (*n* = 12)	680 ± 400 (*n* = 6)	400.86173	400.86134	–0.97	2
2.2	MeO-OH-Tetra-CB	W1	7.796	1.159	80 ± 130 (*n* = 12)	60 ± 60 (*n* = 6)	334.92056	334.92073	0.51	3
2.3	MeO-Tetra-CB Sulfate	X1	6.142	0.914	40 ± 100 (*n* = 11)	20 ± 20 (*n* = 5)	414.87738	414.87899	3.89	2
3.1	OH-Tri-CB	Z1	7.536	1.120	130 ± 170 (*n* = 2)	24 (*n* = 1)	270.94897	270.94888	–0.32	3

a For representative chromatograms and mass spectra of selected metabolites, see Figures S2 to S8.

b RT, retention time in min; RRT, relative retention time to PFOS; ND, not detected.

c The normalized intensity values are the abundance of the analyte relative to the abundance of PFOS per g of tissue for both male and female rats. The values in parentheses indicate the sample number with the detection of the indicated metabolites. Data are presented as the average levels for male and female rats because sex differences in the detection frequency of PCB52 metabolites were only observed for the MeO-Tetra-CB sulfate in the serum.

d δ_*m*/*z*_ ppm values were calculated using the formula: (*mz*_measured_ – *mz*_calculated_)/*mz*_calculated_ × 10^6^.

e Confidence levels were assigned based on the Schymanski framework.^95^ Briefly, metabolites that were identified based on accurate mass, isotope pattern, MS, and MS/MS data have a confidence level of 2. Metabolites that were identified based on accurate mass, isotope pattern, and MS, but not MS/MS, have a confidence level of 3.

### Quality Assurance and Quality Control

All analyses were conducted by using validated standard operating procedures. Surrogate recovery standards were used to ensure the precision and reproducibility of PCB and OH-PCB measurements. Recovery levels of PCB52, 4-OH-PCB52, and X1-OH-PCB52 were corrected for the recoveries of the corresponding recovery standard (Table S4). X1-OH-PCB52 estimated concentrations were calculated based on the relative response factor of 4-OH-PCB52. The detection limits are summarized in Table S5. The precision and recoveries of the analyses were further assessed by measuring the recovery of PCB52 and 4-OH-PCB52 from spiked sample blanks and matched blank tissue matrices (Table S6). For LC-HRMS analyses, 4-sulfoxy-3′-fluoro-4′-chloro-biphenyl ammonium salt (3-F-4′-PCB3 sulfate) was used as a surrogate standard to monitor the recovery rates of the extractions (Table S6). The response of the analytical standards was linear from 1 to 256 ng/mL (Figure S1). PCB metabolite levels were normalized to potassium salt of perfluorooctanesulfonic acid (PFOS), as described.^42,43^ A data set with QA/QC data can be found at DOI: 10.25820/data.006680.^100^

### Statistical Analysis

PCB52 and metabolite levels, adjusted for tissue wet weight, are reported as the mean ± standard deviation. Differences in PCB52 and metabolite levels from the GC-MS/MS analysis were analyzed with the Tobit regression model because some measurements are below the detection limit. To address the variable and occasionally low detection frequency of metabolites in LC-HRMS analysis, logistic regression was employed to investigate potential sex or dose effects. This analysis was conducted for each metabolite, utilizing dichotomized abundance levels classified as either detected or not detected. The statistical analyses were performed with R (version 4.2.3), and *p*-values <0.05 were considered statistically significant.

## Results and Discussion

### PCB52 Tissue Distribution

**Figure 2:**
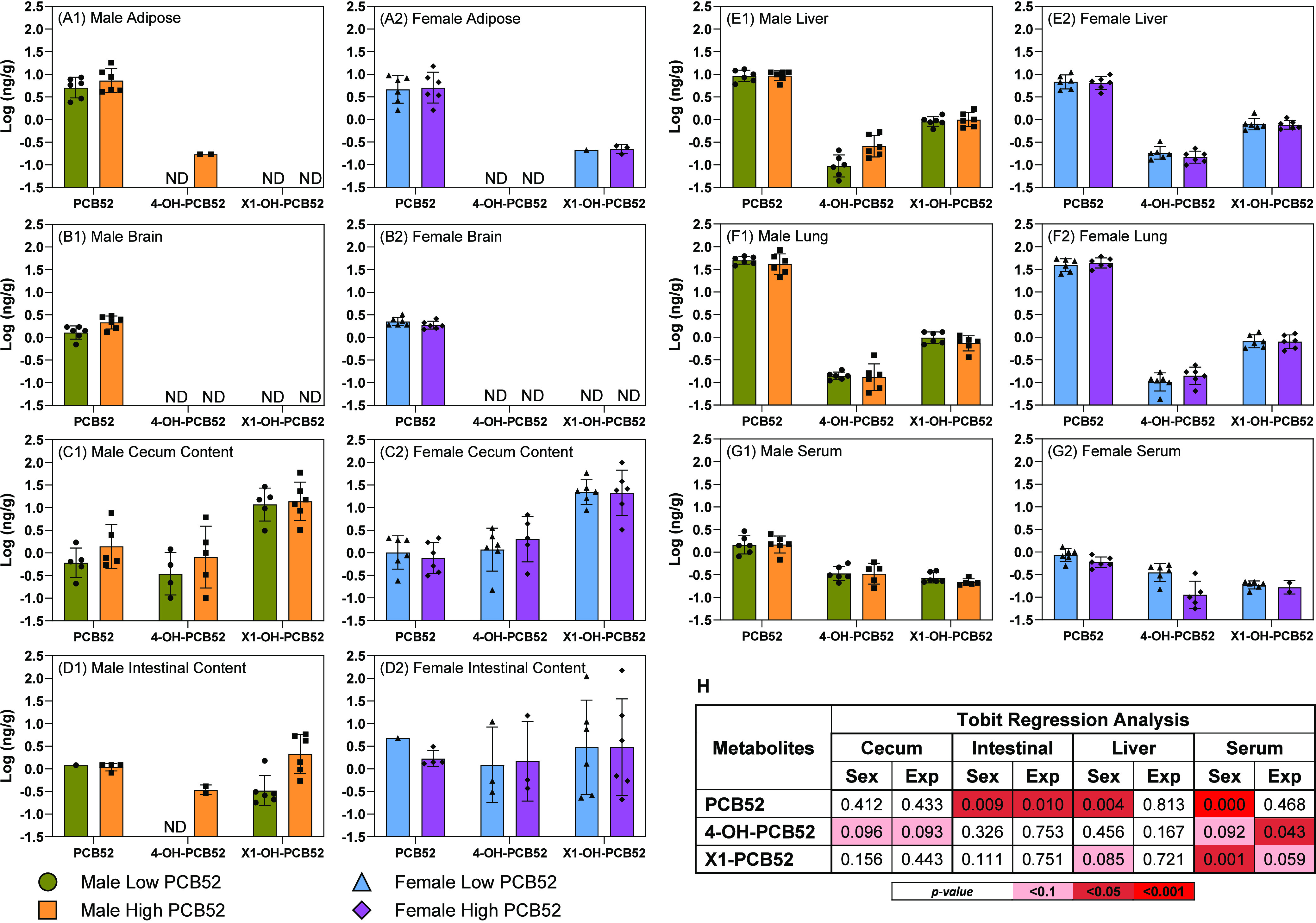
Levels of PCB52 and its monohydroxylated metabolites in (A1) male and (A2) female adipose, (B1) male and (B2) female brain, (C1) male and (C2) female cecum content, (D1) male and (D2) female intestinal content, (E1) male and (E2) female liver, (F1) male and (F2) female lung, and (G1) male and (G2) female serum from rats exposed for 4 h to PCB52 show distinct differences by compartment, sex, and exposure. (H) *P*-values of a Tobit regression analysis to determine differences in PCB analyte levels in cecum content, intestinal content, liver, and serum by sex or exposure. Brain and lung levels were not significantly different by sex and exposure (data not shown). PCB52 and OH-PCB52 metabolite levels were determined by GC-MS/MS. ND, not detected; Exp, exposure group. X1-OH-PCB52, unknown monohydroxylated PCB52 metabolite.

Male rats were exposed to low and high doses of 13 ± 0.4 and 23 ± 1 μg/kg of bw of inhaled PCB52. Female rats inhaled 14 ± 0.4 and 23 ± 2 μg/kg bw PCB52 in the low and high exposure groups. Based on the GC-MS/MS analysis, PCB52 was present in all tissues investigated, including the brain, following these exposures (Figure 2A–G; Table S7). PCB52 was detected in exposed animals with a general trend of decreasing concentration: lung > liver > adipose > brain ≈ intestinal content ≈ cecum ≈ serum, irrespective of sex or dose. Lung concentrations were the highest, as observed in an earlier study in which PCB11 levels similarly followed the rank order lung > serum > liver in tissue from male rats exposed acutely to PCB11 vapors.^26^ It is not surprising that both studies observed the highest PCB levels in the lung, because the lung is the site of PCB absorption. While the lung contained the highest concentration of retained PCB52 in the present acute exposure study, repeated dose inhalation studies report different rank orders in PCB tissue levels, with the highest PCB levels observed in the adipose tissue due to the redistribution of PCBs to the adipose tissue and their (hepatic) metabolism.^12,25,44^

Notably, PCB52 was detected in the brain (Figure 2F). The transport of PCB52 to the brain likely occurred through passive diffusion across the blood-brain barrier.^45^ Alternatively, PCB52 may have crossed the blood-brain barrier bound to the transport protein transthyretin (TTR).^1,17,18,46^ Because PCB52 is a potential neurotoxicant, we calculated the neurotoxic equivalent quotient (NEQ) levels for PCB52 applying published neurotoxic equivalent factors for multiple modes of action to tissue residue levels.^47^ The NEQ levels of PCB52 in the brain ranged from 0.3 to 1.3 ng/g across all exposure groups. For comparison, the average NEQ level was 29 ng/g in the brain of postnatal day 21 mice developmentally exposed to PCB95 (0.1 to 6.0 mg/kg bw) via the maternal diet.^48^ The dosing paradigm in the earlier PCB95 disposition study is associated with neurotoxic outcomes in rodents.^49−51^ The median ΣNEQ levels in post-mortem human tissue samples were 290 pg/g for older and 7 pg/g for younger donors.^19^ Thus, the NEQ levels of PCB52 in the rat brain following acute inhalation exposure fall within the NEQ range observed in human brain samples and are 22-fold lower than the NEQ levels associated with neurotoxic outcomes in the mouse brain following repeated dose developmental PCB exposure.

PCB52 in the gastrointestinal (Figure 2C–D) tract likely originated from PCB52 swallowed following mucociliary clearance of inhaled PCB52 from the lung, consistent with a study of PCB11 disposition following intratracheal instillation.^24^ Dose effects on PCB52 tissue levels were observed only in the intestinal content (Figure 2H) despite the 1.6-fold difference in the estimated PCB52 dose. The lack of a dose effect on PCB52 tissue levels can be attributed to experimental errors in the dose determination, variability of PCB levels between animals, and subtle differences in the time course of PCB52 tissue levels at the low vs high dose. Interestingly, PCB52 levels showed a sex effect in the intestinal content (Figure 2H), likely because of sex differences in physiological parameters, such as the gastrointestinal transit time.^52^ Moreover, a significant effect of sex on PCB52 levels was observed in the liver and serum, with higher PCB52 levels in male rats than female rats (Figure 2H). Although the estimated PCB52 doses differed significantly by sex (Figure 1B), the differences are small and unlikely to explain the sex differences in PCB52 tissue levels. Alternatively, sex differences in the cytochrome P450 (CYP) expression in the lung or liver may contribute to sex differences in the PCB52 levels.^53^

### Targeted GC-MS/MS Analysis of Monohydroxylated PCB52 Metabolites in Tissues

Cytochrome P450 enzymes oxidize PCB52 to OH-PCB isomer mixtures.^18^ These OH-PCBs can be neurotoxic,^18,54,55^ for example, by mechanisms involving the ryanodine receptor.^35,56^ 4-OH-PCB52, a human-relevant metabolite,^57^ and one unknown OH-PCB metabolite, X1-OH-PCB52, were present in the rat liver, lung, and serum following inhalation exposure to PCB52 vapors (Figure 2). No OH-PCB metabolites were detected in the brain by GC-MS/MS. Based on the retention time order, X1-OH-PCB52 likely corresponds to 2,2′,5,5′-tetrachlorobiphenyl-3-ol (3-OH-PCB52), a *meta*-hydroxylated PCB52 metabolite.^58,59^ Metabolism studies with rat liver microsomes also identified 4-OH-PCB52, 3-OH-PCB52, and a secondary metabolite, 3,4-diOH-PCB52, as oxidation products of PCB52.^60−62^ Further studies with an authentic analytical standard are needed to confirm the tentative identification of X1-OH-PCB52 as 3-OH-PCB52. Hydroxylated PCB52 metabolites have not been reported in human samples, consistent with rapid metabolism (e.g., sulfation) or excretion of the PCB52 metabolites. However, various OH-PCB congeners can be found in human blood, mostly congeners with five to eight chlorine substitutes.^63,64^

Although OH-PCB levels in adipose tissue are rarely reported in the literature, there is evidence that PCB52 metabolites alter the transcriptome of human preadipocytes in culture more drastically than PCB52.^65^ Our results show that in both exposure groups X1-OH-PCB52 was detected in the adipose tissue of female but not male rats (Figure 2A; Table S7). After IP exposure to PCB52 in female rats, 4-OH-PCB52 was only detected in the adipose from one high-exposure animal.^66^ Furthermore, one study found traces of a meta-hydroxylated PCB metabolite in the adipose tissue in rats following subacute oral exposure to PCB95.^67^ Other disposition studies detected OH-PCBs in the liver and serum but not the adipose tissue of mice exposed orally to repeated doses of PCB91 or PCB95,^48,68,69^ likely because the analytical method used by this earlier study lacked sensitivity. The evidence that OH-PCBs are present in human adipose and liver tissues is also limited. One study from Sweden reported the presence of PCB metabolites, including OH-PCBs, in human adipose and liver samples.^70^

Because they are potential neurotoxicants, it is notable that OH-PCB52 metabolites are below the detection limit (0.16 ng/g for 4-OH-PCB52) in the brain following acute inhalation of PCB52, irrespective of sex and exposure group (Figure 2B; Table S7). Only a few studies have investigated the presence of PCB metabolites in the brains of mammals following acute exposure; however, there is evidence that PCB metabolites are present in the brains following longer, repeated exposures. After intraperitoneal exposure to a synthetic PCB mixture, OH-PCBs were detected in the brains of dogs.^71^ Similarly, X1-OH-PCB52 and 4-OH-PCB52 were detected with a detection frequency of 20% in the rat brain 3 weeks after an intraperitoneal administration of a high PCB52 dose (100 mg/kg bw).^66^ PCBs and OH-PCBs have also been detected in the brain of *Macaca fuscata*, with levels ranging from 6.7 to 31 pg/g,^72^ and in a small number of post-mortem human brain tissue samples.^19^ While OH-PCBs have been reported in the brains of exposed mammals following longer repeated exposures, our study indicates acute exposures may not be sufficient for metabolizing and detecting OH-PCBs in the brain.

We detected 4-OH-PCB52 and X1-OH-PCB52 in the cecum and intestinal contents of rats following PCB52 inhalation (Figures 2C–D; Table S7). Notably, the levels of the OH-PCB52 metabolites were higher in the intestinal content than in tissues from the PCB52-exposed rats. X1-OH-PCB52, the putative meta-hydroxylated metabolite, was the major metabolite in the cecum and intestinal content. Similarly, meta-hydroxylated metabolites were the primary fecal metabolites in mice orally exposed to PCBs.^41,69^ The observation that high levels of OH-PCB52 metabolites are detected in the intestinal content after inhalation exposure is consistent with a disposition study with intratracheally instilled ^14^C-labeled PCB11.^24^ This study found that PCB11 exposure resulted in radioactivity in the systemic circulation within 12 min. However, it took anywhere from 50 to 200 min for radioactivity to show up in the lower gastrointestinal tract. The highest levels of extractable PCB11 metabolites were observed in the jejunum and ileum approximately 100 min postexposure. The presence of PCB52 metabolites in the intestinal content could result from oxidative PCB52 metabolism in the lung or liver after its absorption into systemic circulation, followed by the excretion of resulting metabolites by the liver. Additionally, inhaled PCB52 can be eliminated through the mucociliary escalator, absorbed in the gastrointestinal tract, and metabolized in the liver, as has been suggested for PCB11 instilled intratracheally.^24^

### LC-HRMS Analysis of PCB52 Metabolites

**Figure 3:**
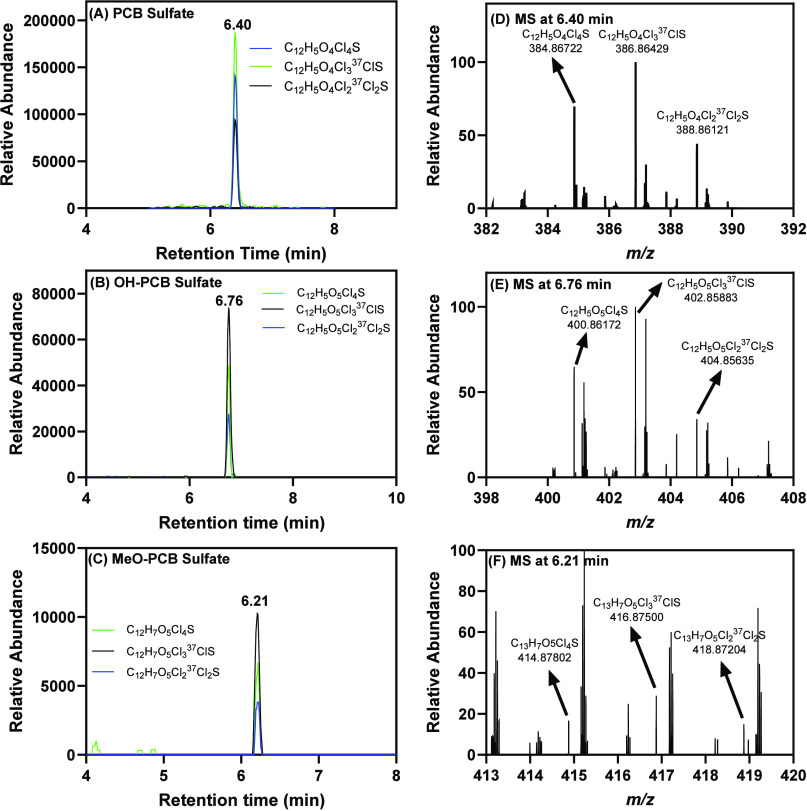
Representative chromatograms extracted based on the theoretical accurate mass of high-abundance isotope ions show the presence of (A) one PCB sulfate (chromatogram in green, [C_12_H_5_Cl_4_O_4_S]^−^, *m*/*z* 384.86681 for the monoisotopic ion) 6.40 min, (B) one OH-PCB sulfate (chromatogram in green, [C_12_H_6_Cl_4_O_5_S]^−^, *m*/*z* 400.86173 for the monoisotopic ion) at 6.76 min, and (C) one MeO-PCB sulfate (chromatogram in green, [C_13_H_7_Cl_4_O_5_S]^−^, *m*/*z* 414.87738 for the monoisotopic ion) at 6.21 min. The accurate masses of the isotope ions of (D) the PCB sulfate eluting at 6.40 min, (E) the OH-PCB sulfates eluting at 6.76 min, and (F) the MeO-PCB sulfate eluting at 6.21 min match the theoretical accurate mass and isotopic pattern of a tetrachlorinated compound. The LC-HRMS analysis was performed in the negative polarity mode. For chromatograms and MS data of other metabolites, see the Supporting Information.

**Figure 4:**
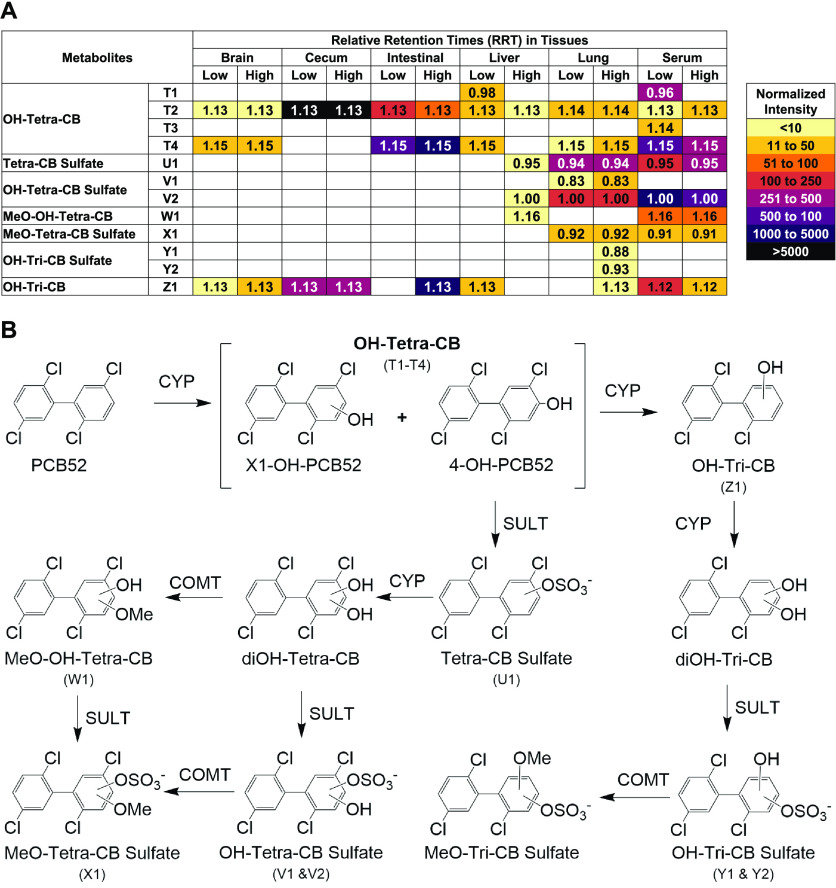
PCB52 metabolite profiles observed in (A) different compartments suggest (B) the metabolism pathway for inhaled PCB52. (A) A heatmap-style summary of the LC-HRMS results across exposure groups and tissues reveals differences in the PCB52 metabolite profiles across tissues at the time point investigated. The metabolites were identified and matched across different tissues based on their relative retention times (RRT). The intensity of each metabolite is represented by a color gradient, which has been normalized to facilitate the comparison across exposure groups and tissues. (B) Proposed metabolism scheme of PCB52 in adolescent rats following a 4 h inhalation exposure to PCB52. The metabolism pathways are based on the metabolites detected in the targeted GC-MS/MS and the LC-HRMS analyses; see text for details. CYP, cytochrome P450 enzyme; SULT, sulfotransferase; COMT, catechol-*O*-methyltransferase.

Following PCB exposure, various metabolites other than OH-PCBs, such as PCB sulfate and other conjugates, can be formed in rats. These metabolites can also have detrimental effects on the brain.^18^ Therefore, we employed a published LC-HRMS technique^42,43,73^ to comprehensively examine the PCB52 metabolites in selected tissues following acute PCB52 inhalation. The objective was to elucidate the metabolic pathway of PCB52 in rats after the inhalation of PCB52. The LC-HRMS analysis identified several PCB52 metabolites in the liver (7 metabolites), lung (9 metabolites), and serum (9 metabolites), including sulfated, methoxylated, and dechlorinated metabolites (Table 1; Figures 3 and 4). Representative chromatograms and mass spectra showing the presence of one PCB sulfate, one OH-PCB sulfate, and one MeO-PCB sulfate in the lung are shown in Figure 3; for chromatograms and mass spectra of other PCB52 metabolites in other compartments, see Figures S2–S8. It is noteworthy that the metabolite profiles detected in tissues and serum differ between IP administration vs inhalation of PCB52.^66^ For example, 6 of 11 metabolites detected in the livers of rats exposed IP to PCB52 were not observed after inhalation exposure, whereas 2 of 7 metabolites were unique following PCB52 inhalation. Additional studies are needed to determine whether these differences are due to the route of exposure or reflect differences in the PCB52 dose and exposure time.

Three peaks corresponded to hydroxylated metabolites, but no other PCB52 metabolites were detected in the brain. This finding contrasts with the results from the targeted GC-MS/MS analysis, possibly because the corresponding metabolites are outside the retention time windows used for the GC-MS/MS analysis. More generally, analytical standards are needed to identify hydroxylated PCB metabolites observed in LC vs GC analyses.^74^ Cecum and intestinal contents contained two and three hydroxylated metabolites, respectively. In contrast, complex mixtures of sulfated or methoxylated mono- to dihydroxylated PCB metabolites were detected by LC-HRMS in the feces of mice following subacute oral exposure to a PCB mixture.^43^ This discrepancy is likely due to the short exposure time in our inhalation study. The detection frequency (DF) of the metabolites varied across exposure groups and tissues and ranged from nondetected to 100%. Based on logistic regression, the tetra MeO-PCB sulfate detection frequency in serum was marginally and significantly affected by sex and dose, respectively (Table 1).

It is widely known that MeSO_2_-PCBs accumulate in the lungs;^75,76^ however, little is known about the levels and profiles of OH-PCBs and their conjugates in the lung.^26^ Moreover, it is unknown whether PCBs are metabolized in the lung following inhalation exposure. For example, CYP2B enzymes, which are involved in the metabolism of multiple ortho-substituted PCB congeners, are active in the lung.^26,77^ SULT1A1, which is known to sulfate OH-PCBs,^78,79^ is expressed in the rat lung.^80^ There is also evidence that PCB metabolites alter the metabolism of xenobiotics in the rat lung.^81^ Thus, PCB52 may be metabolized in the lungs following inhalation exposure. Consistent with the putative metabolism of inhaled PCB52 in the lung, the metabolite profile in the lung appeared more complex than in the other compartments investigated (Figure S9; Table 1). We detected two OH-PCB52 metabolites in the lung (detection frequency [DF] > 66%), one PCB sulfate (DF > 83%), two hydroxylated PCB sulfate (DF from 8 to 100%), and one methoxylated PCB sulfate (DF > 83%). One dechlorinated OH-PCB (OH-Tri-CB) was present in the lung (DF 8%). In addition, two dechlorinated OH-PCB sulfates (OH-Tri-CB sulfate) were detected in the lungs of the high-dose exposure group (DF from 17 to 42%). This sulfated metabolite is likely produced from an OH-PCB by replacing a chlorine substituent with a hydroxyl group, followed by sulfation of the dechlorinated dihydroxy metabolite.^31^

Multiple metabolites were discovered in both the liver and the serum (Figure S9; Table 1). The relative levels of these metabolites were higher in the serum (Figure 4A). Three and four peaks corresponding to OH-PCB52 metabolites were observed in the liver (DF from 8 to 33%) and serum (DF from 8 to 50%), respectively. We also found the OH-Tri-CB present in the lung but with detection frequencies of ≤25%. In contrast, we detected only two OH-PCB52 congeners in the GC-MS/MS analysis, likely because some OH-PCBs were not captured by the targeted GC-MS/MS method or were not derivatized with diazomethane.^82^ One PCB sulfate was found in the liver (DF 8%) and serum (DF > 58%). Similarly, PCB sulfates have been detected in the liver and serum of rodents following acute inhalation exposure to PCB3^28,83^ or oral exposure to PCB11,^73^ as well as in the serum of human populations.^84,85^

The LC-HRMS analysis also identified sulfated, methoxylated, or dihydroxylated PCB52 metabolites in the liver and serum (Figure S9; Table 1). The OH-Tetra-CB sulfate found in the lung was also detected in serum and liver (DF > 50% and 58%, respectively). A second OH-Tetra-CB sulfate was also found in the lung (DF 8 to 33%). Hydroxylated PCB sulfates have been detected in rat urine following inhalation of PCB3^83^ and in the liver and serum of mice orally exposed to PCB11.^73^ These metabolites can be formed by the sulfation of PCB catechol metabolites,^83^ which are formed in a two-step oxidation process by rat cytochrome P450 enzymes.^86,87^ Alternatively, PCB sulfates can be oxidized to OH-PCB sulfates, as shown after intravenous injection of a PCB11 sulfate in rats.^88^ Both metabolic pathways also contribute to the formation of OH-PCB sulfates in cell culture models, as shown for PCB3.^30^ Moreover, one methoxylated OH-PCB was detected in the liver and serum (DF 42% and 50 to 100%, respectively), and one methoxylated PCB sulfate (DF 42 to 92%) was present in the serum. These metabolites are likely formed via the methylation of PCB52 catechols by catechol-*O*-methyltransferases (COMT). These metabolites have also been observed in cell culture^29−31^ and animal studies.^43,89,90^ However, the disposition and toxicity of methoxylated PCB metabolites have received little attention.

### Proposed Metabolism Pathway of PCB52 in Adolescent Rats

Based on the metabolites detected in this study, we propose a metabolic pathway of PCB52 in adolescent rats after inhalation exposure (Figure 4B). Initially, PCB52 is oxidized by cytochrome P450 enzymes in the lung or liver to monohydroxylated OH-PCB metabolites (i.e., 4-OH-PCB52 and X1-OH-PCB52). This oxidation may involve a 3,4-arene oxide intermediate or occur by directly inserting an oxygen atom into the aromatic C–H bond, as suggested by *in vitro* metabolism studies using rat liver microsomes.^60−62^ Cytochrome P450 enzymes further oxidize the OH-PCB metabolites or the corresponding conjugates to di- and trihydroxylated PCB metabolites. These metabolites are metabolized to corresponding sulfates and methoxylated PCB metabolites by sulfotransferases (SULT) or COMT. Also, OH-PCB metabolites undergo chlorine displacement to form dechlorinated dihydroxylated PCB metabolites that undergo further biotransformation to sulfate conjugates and methoxylated PCBs, as reported.^66,88^ This metabolism pathway is consistent with published *in vitro* and *in vivo* studies^18,91^ and comparable to our previous work in rats intraperitoneally exposed to PCB52.^66^ Importantly, the complex PCB52 metabolite profile observed in the lung is suggestive of the first-path metabolism of PCB52 in the lung. This possibility warrants further investigations considering the significant contribution of inhalation to human exposures to PCBs.^10^

### Sex Differences in the Disposition of Inhaled PCB52

The study revealed sex differences in the overall exposure levels, metabolite formation, and PCB and metabolite distribution patterns. As discussed, adolescent males retained significantly more PCB52 in the liver and serum in comparison to exposed females. The levels of 4-OH-PCB52 and X1-OH-PCB52 were higher in the cecum and intestinal contents of female rats than male rats exposed to PCB52; however, this difference was not statistically significant. These differences in the excretion of PCB52 suggest that there may be sex differences in the toxicokinetics of inhaled PCB52 in adolescent rats that warrant further investigation. Such differences could be due to sex differences in the levels of drug-metabolizing enzymes, such as cytochrome P450 enzymes, in the liver or lung^53^ or the gastrointestinal transit time.^52^ Studies with PCB136, a PCB congener structurally related to PCB52, showed that this congener is oxidized in a sex-dependent manner by precision-cut rat liver tissue slices. Oral dosing studies in rodent models reported conflicting results, with most studies not observing sex differences in the disposition of PCBs and their metabolites.^50,51,92,93^ Thus, the sex-dependent metabolite formation following PCB52 inhalation has implications for PCB52 neurotoxicity because OH-PCBs can affect cellular targets relevant to PCB neurotoxicity.^94^ Further studies are needed to identify the absorption, distribution, metabolism, and elimination processes that contribute to sex differences in the toxicokinetics of PCB52 metabolites following the inhalation of PCB52.

### Environmental and Human Health Implications

The study addresses the misconception that the diet is the major route of PCB exposure in humans^7^ by demonstrating that, consistent with a small number of earlier nose-only inhalation studies,^12,13,25^ PCB52 is readily absorbed after inhalation. Our findings demonstrate for the first time that PCB52 inhalation leads to the formation of complex PCB52 metabolite profiles, including potentially toxic sulfated, methoxylated, and dechlorinated PCB52 metabolites, primarily in the liver, lung, and serum of adolescent rats. Because PCBs are still present in building materials and are found in the indoor air of schools and residential buildings, the detection of potentially toxic metabolites in target tissues in this study raises concerns about chronic inhalation exposure to these compounds, especially among sensitive populations like adolescents. By characterizing the metabolites formed following acute PCB52 inhalation, the study highlights the need for developing toxicokinetic models and performing toxicity assessments of inhaled PCBs, such as PCB52, and their metabolites relevant to indoor environments, especially following subacute to chronic inhalation exposure. Regulatory agencies urgently need such studies to assess the risks associated with human exposure to inhaled PCB.

## Supporting Information Available

Descriptions of the sample preparation, quality assurance/quality control, body and organ weights, PCB52 or metabolite levels, and LC-HRMS results (PDF)
