# Supplementary material for: Distribution of 2,2′,5,5′-Tetrachlorobiphenyl (PCB52) Metabolites in Adolescent Rats after Acute Nose-Only Inhalation Exposure
Source: Environ Sci Technol. 2024 Mar 28;58(14):6105–16. doi: 10.1021/acs.est.3c09527 (PMC11008251; doi:10.1021/acs.est.3c09527)
Supplement: Supplementary file 1 — es3c09527_si_001.pdf [file es3c09527_si_001.pdf]

SUPPORTING INFORMATION

Distribution of 2,2',5,5'-Tetrachlorobiphenyl (PCB52) Metabolites in Adolescent Rats After Acute Nose-Only Inhalation Exposure

*Amanda J. Bullert[1,2,†], Xueshu Li[1,†], Binita Gautam[1], Hui Wang[1], Andrea Adamcakova-Dodd[1], Kai Wang[3], Peter S. Thorne[1,2], Hans-Joachim Lehmler[1,2]**

[1]Department of Occupational and Environmental Health, The University of Iowa, Iowa City, Iowa 52242, United States. [2]Interdisciplinary Graduate Program in Neuroscience, The University of Iowa, Iowa City, Iowa 52242, United States. [3]Department of Biostatistics, University of Iowa, Iowa City, IA 52242, USA.

*Corresponding Author:
Dr. Hans-Joachim Lehmler
The University of Iowa
Department of Occupational and Environmental Health
University of Iowa Research Park, B164 MTF
Iowa City, IA 52242-5000
Phone: (319) 335-4981
Fax: (319) 335-4290

Number of Pages: 29

Number of Tables: 8

Number of Figures: 9

[†] Both authors contributed equally to the manuscript

Table of Contents

Chemicals S3

Extraction of PCB52 and its hydroxylated metabolites for targeted gas chromatography-tandem mass spectrometry (GC-MS/MS) S3

GC-MS/MS analyses S5

Quality assurance and quality control for the GC-MS/MS analyses S5

Extraction of PCB metabolites from tissues for liquid chromatography-high resolution mass spectrometry (LC-HRMS) analysis S6

LC-HRMS analysis S7

Processing of LC-HRMS Orbitrap data and figure visualization S8

Table S1. Abbreviations and unique identifiers of the test compounds and analytical standards S9

Table S2. Summary statistics of body and organ weights S10

Table S3. Summary of organ weights adjusted for body weight S11

Table S4. Recoveries (%) of the Ongoing Precision and Recovery standard spiked into matrices from naïve animals in the GC-MS/MS analysis S12

Table S5. Method detection limits (ng) and limits of detection for each tissue type (ng/g) for the gas chromatographic quantifications of PCB52 and 4-OH-PCB52 S13

Table S6. Surrogate standard recoveries (%) from all tissues in the GC-MS/MS analysis S14

Table S7. PCB or metabolite ng/g wet weight detected levels of each compound across tissue and exposure group S15

Table S8. Precursor ions, product ions, and collision energies for each analyte included in the GC/MS/MS analysis S16

Figure S1. Calibration curve of (A) PFOS, (B) 3-F-4'-PCB3 sulfate, (C) 4-OH-PCB52 and (D) 4-PCB52 sulfate S17

Figure S2. One trichlorinated OH-PCB metabolite was detected by LC-Orbitrap MS in the intestinal content of PCB52-exposed rats S18

Figure S3. One tetrachlorinated OH-PCB metabolite was detected by LC-Orbitrap MS in the intestinal content of PCB52-exposed rats S19

Figure S4. One tetrachlorinated PCB sulfate was detected by LC-Orbitrap MS in the lung of PCB52-exposed rats S20

Figure S5. One trichlorinated OH-PCB sulfate was detected by LC-Orbitrap MS in the serum of PCB52-exposed rats S21

Figure S6. One tetrachlorinated OH-PCB sulfates was detected by LC-Orbitrap MS in the liver of PCB52-exposed rats S22

Figure S7. One tetrachlorinated MeO-OH-PCB was detected by LC-Orbitrap MS in the serum of PCB52-exposed rats S23

Figure S8. One tetrachlorinated MeO-PCB sulfate was detected by LC-Orbitrap MS in the serum of PCB52-exposed rats S24

Figure S9. The relative levels of PCB52 metabolites from LC-HRMS show distinct differences by compartment but not sex in rats exposed for 4 h to PCB52 S25

References S27

Chemicals. The test compound, PCB52, was synthesized and authenticated using a published guideline.[1] Details regarding its authentication are reported elsewhere.[2, 3] Analytical standards for gas chromatographic analyses, including 3,3',4,4'-tetrachlorobiphenyl (PCB77) and 2,5,2',5'-tetrachlorobiphenyl-4-ol (4-OH-PCB52) were synthesized and authenticated as described previously.[3-6] 2,3,3',4,5,5'-Hexachlorobiphenyl-4'-ol (4'-OH-PCB159) and 2,2',3,4,4',5,6,6'-octachlorobiphenyl (PCB204) were purchased from AccuStandard, Inc (New Haven, CT). PCB77 and 4'-OH-PCB159 were added to all samples as surrogate recovery standards. PCB204 was used as an internal standard to adjust for volume differences between samples. The recovery standard for the LC-HRMS analysis, 4-sulfooxy-3'-fluoro-4'-chloro-biphenyl ammonium salt (3-F-4'-PCB3 sulfate) was prepared and authenticated as reported elsewhere.[7, 8] The potassium salt of perfluorooctanesulfonic acid was provided by Thermo Fisher Scientific (Pittsburg, PA).

Extraction of PCB52 and its hydroxylated metabolites for targeted gas chromatography-tandem mass spectrometry (GC-MS/MS). PCB52 and its hydroxylated metabolites were extracted with a liquid-liquid extraction protocol from adipose (0.11 ± 0.01 g), brain (0.7 ± 0.08 g), liver (0.5 ± 0.01 g), and lung tissue (0.5 ± 0.01 g), as described.[9, 10] Briefly, tissues were homogenized with 3 mL of isopropanol and 1 mL of diethyl ether. Tissue samples were spiked with surrogate recovery standards (20 ng of PCB77 in 200 µL of isooctane and 20 ng of 4'-OH-PCB159 in 200 µL of methanol). The samples were capped and inverted for 5 min at 40 rpm and centrifuged at 1,690 g for 5 min to facilitate phase separation. The organic phase was transferred to a second tube containing 5 mL of 0.1 M phosphoric acid in 0.9% NaCl solution. The tissue pellets were resuspended with 1 mL of isopropanol and 2.5 mL hexanes-diethyl ether (9:1, v/v). The samples were vortexed, inverted, and centrifuged as described above. The organic phases were

removed and combined with those from the first extraction step. Next, the aqueous phases were re-extracted with 3 mL hexanes-diethyl ether (9:1, v/v), and the organic phases were combined and concentrated to approximately 0.5 mL under a gentle stream of nitrogen.

Five drops of methanol and 0.5 mL of diazomethane (about 5 mmol) in diethyl ether were added to the organic extracts.[9] All samples were stored at 4°C for at least 3 h to allow enough time for derivatization. Excess diazomethane was evaporated under a gentle stream of nitrogen in a fume hood. The extracts were reconstituted in 0.5 mL of hexanes and loaded onto glass SPE cartridges containing 0.2 g of activated silica gel (bottom) and 2 g of acidified silica gel (silica gel: H_2SO_4, 2 :1, w/w; top). PCBs and OH-PCBs (as methylated derivatives) were eluted with 14 mL of dichloromethane. The eluent was concentrated to near dryness, and the solvent was exchanged for hexanes. The extracts (~3 mL in hexanes) were treated with 4 mL of concentrated sulfuric acid, concentrated to near dryness, and spiked with 20 ng of internal standard (PCB204 in isooctane, 200 µL) for gas chromatographic analysis. Blank samples and an ongoing precision and recovery standard were analyzed in parallel with all samples.

Serum (0.5 ± 0.11 g), intestinal content (0.06 ± 0.01 g), and cecum content (0.51 ± 0.02 g) were processed using the following extraction protocols. For serum, 3 mL of aqueous 1% KCl solution was added to each sample, followed by the surrogate recovery standards (20 ng of PCB77 in 200 µL of isooctane and 20 ng of 4'-OH-PCB159 in 200 µL of methanol). Intestinal and cecum content samples were homogenized in 3 mL of 0.2 M sodium acetate buffer (pH 5) and spiked with the surrogate recovery standards (20 ng of PCB77 in 200 µL of isooctane, 4'-OH-PCB159 in 200 µL of methanol, and 3-F-4'-PCB3 sulfate in DMSO). Samples underwent incubation with 50 µL sulfatase (type H-2 from *Helix pomatia*, Sigma-Aldrich, Burlington, MA) for 16 h at 37°C in a shaking water bath. Subsequently, 1 mL of 6 M HCl, 5 mL of 2-propanol, and 5 mL of 1:1

hexanes-MTBE mixture (v/v) was added to the serum, intestinal, and cecum content samples. The samples were inverted for 5 min and centrifuged at 1,690 g for 5 min to facilitate the phase separation. Next, the organic phases of each sample were transferred to new glass tubes, and the aqueous phases were re-extracted with 3 mL of hexanes. Next, 3 mL of aqueous 1% KCl was added to the combined organic extracts, and samples were inverted and centrifuged as described above. The organic phase was transferred to a new tube, and the aqueous KCl phase was re-extracted with 3 mL hexanes. The combined organic extracts from the samples were evaporated to near dryness, diluted with 0.5 mL hexanes, and derivatized and cleaned up as described for tissues. Blank samples and an ongoing precision and recovery standard were processed in parallel with all samples.

GC-MS/MS analyses. PCB52 and metabolite determinations were conducted using the multiple reaction monitoring setting (MRM) on an Agilent 7890 A GC system equipped with an Agilent 7000 Triple Quad and Agilent 7693 autosampler. Gas chromatographic separations were performed with an SPB-Octyl capillary column (30 m length, 25 mm inner diameter, 0.25 μm film thicknesses: Supelco, Bellefonte, PA); see **Table S8** for the precursor ions, product ions, and collision energies for each analyte. Samples were injected in the solvent vent injection mode with a helium (carrier gas) flow of 0.75 mL/min. Nitrogen was used as the collision gas. The following temperature program was used for the separation of PCB52 and its metabolites: Initial temperature of 45°C, hold for 2 min, 100°C/min to 75°C, hold for 5 mins, 15°C/min to 150°C, hold for 1 min, 2.5°C/min to 280°C, and final hold of 5 min. The unknown OH-PCB was quantified using the relative response factor of 4-OH-PCB52.

Quality assurance and quality control for the GC-MS/MS analyses. All analyses were performed following established Standard Operating Procedures. Appropriate blank tissue samples

were extracted and analyzed in parallel. Surrogate recovery standards (i.e., PCB77, 4'-OH-PCB159, and 3-F-4'-PCB3 sulfate) were spiked into every sample immediately prior to extraction to correct for analytical losses during sample workup and assess the precision and reproducibility of the extraction across the entire study. Average OH-PCB recovery rates, including the range of recoveries and relative standard deviation for each standard, are provided in **Table S2**. The method detection limit (MDL) was calculated from method blanks with the formula:[11, 12]

$$MDL = \bar{x} + t_{(n-1,\ 1-\alpha=0.99)} \times SD,$$

where $\bar{x}$ is the mean of the replicates from the method blanks, $t_{(n-1, 1-\alpha=0.99)}$ is the Student's t-test value for the n – 1 degree of freedom with 99% confidence level, and SD represents the standard deviation of the replicates.

Extraction of PCB metabolites from tissues for liquid chromatography-high resolution mass spectrometry (LC-HRMS) analysis. Aliquots of brain (213-338 mg, n=38), cecum (7-33 mg, n=34), intestinal content (25-56 mg, n=38), liver (247-260 mg, n=38), lung (251-258 mg, n=38) and serum (50-283 mg, n=34) were analyzed by LC-HRMS to identify PCB52 metabolites using a modified protocol based on previous studies.[8] Tissues were homogenized in a glass tube with 2 mL of Milli-Q water using a TissueRuptor (Qiagen). The homogenates were then spiked with surrogate standards, 3-F-4'-OH-PCB3 and 3-F-4'-PCB3 sulfate (50 ng of each), in acetonitrile. The homogenate was vortexed for 10 s, and 4 mL of acetonitrile with 1% formic acid was added. After adding 200 mg of sodium chloride and 800 mg of magnesium sulfate, the samples were shaken vigorously, inverted for 5 min, and centrifuged at 1181 g for 5 min to facilitate phase separation. The organic phase on the top was passed through hybrid phospholipid solid-phase extraction (Hybrid SPE) cartridges (3 mL, Millipore Sigma, Burlington, MA), which were loaded

with 3 g of a mixture of anhydrous sodium sulfate and anhydrous magnesium sulfate (1:1, w/w) and preconditioned with 3 mL of acetonitrile. The aqueous phase was reextracted with an additional 1 mL of acetonitrile, and the organic phase was again passed through the Hybrid SPE cartridge. The Hybrid SPE cartridges were washed with 3 mL of acetonitrile. The combined eluents were evaporated to dryness using a Savant SpeedVac SPD vacuum concentrator with an RVT5105 refrigerated vapor trap (Thermo Scientific, Waltham, MA) at 35°C. The residual samples were redissolved in 300 μL of acetonitrile and transferred to microcentrifuge tubes. Potassium perfluorooctanesulfonate (PFOS, 50 ng in acetonitrile) was spiked to the samples as a volume corrector. After the solvent was evaporated to dryness using a SpeedVac concentrator, the extracts were reconstituted with 200 μL of mobile phase (H_2O-ACN-MeOH = 20-40-40, volume %) and kept in a -20°C freezer for 30 min. The extracts were vortexed for 10 s and centrifuged for 10 min at 4°C and 16,000 g to precipitate the protein. The supernatant was transferred to another microcentrifuge tube and centrifuged using the same parameters. The supernatants were transferred to autosampler vials and kept at -80°C until LC-HRMS analysis.

LC-HRMS analysis. Extracts were analyzed at the High-Resolution Mass Spectrometry Facility at the University of Iowa on a Q-Exactive Orbitrap mass spectrometry (Thermo Fisher Scientific) with an AXQUITY UPLC-C18 column (particle size: 1.7 μM, 2.1 × 100 mm, Waters, Milford, MA, USA). Mobile phases A and B were water and acetonitrile with a 0.3 mL/min flow rate. The pressure range of the chromatographic system was 4000 to 8000 psi. The UHPLC gradient program was as follows: start at 5% B, hold for 1 min, increase linearly to 95% B, hold for 3 min, return to 5% B, and hold for 4 min before the next injection. The injection volume was 2 μL. The mode used on the Q-Exactive Orbitrap Mass Spectrometry was negative polarity. The current and spray voltage were 18.2 μA and 2472 V. The gas flow rate of the auxiliary and sheath

were 2 mL/min and 48 mL/min, respectively. The auxiliary and capillary temperatures were 413°C and 256°C, respectively. The analyses were performed in the full scan mode with a range of 85 to 1000 *m/z*. The full scan resolution setting was 70,000, the autogain control target setting was $1x10^6$, and the maximum interval time (IT) was 200 ms.

Processing of LC-HRMS Orbitrap data and figure visualization. The suspected peaks of PCB metabolites were extracted from the acquired data (as .raw file) with Themo Xcalibur (version 4.3, Thermo Fisher Scientific). A tolerance of 5 ppm, smoothing factor of 7, and mass precision decimals of 5 were used to extract accurate mass data and relative peak areas. The isotopic pattern of chlorine was closely examined to confirm chlorine compounds, as described. Peak areas for each metabolite, determined for the most abundant isotopic peak, were normalized to the peak area of PFOS.

Table S1. Abbreviations and unique identifiers of the test compounds and analytical standards used in this study.

Abbreviation	IUPAC Name	FORMULA	Isomeric SMILES	InChI	InChIKey	CAS Registry Number	CAS Registry URL	PubChem CID	PubChem Link	DTXSID	Comptox Link
3-F-4'-OH-PCB3	4-(4-chloro-3-fluorophenyl)phenol	C12H8ClFO	C1=CC(=CC=C1C2=CC(=C(C=C2)Cl)F)O	InChI=1S/C12H8ClFO/c13-11-6-3-9(7-12(11)14)8-1-4-10(15)5-2-8/h1-7,15H	DBNVNCYZHMXJJL-UHFFFAOYSA-N	893736-99-7	https://commonchemistry.cas.org/detail?cas_rn=893736-99-7	20099942	https://pubchem.ncbi.nlm.nih.gov/compound/20099942	NA	NA
3-F-4'-PCB3 sulfate	4-sulfooxy-3'-fluoro-4'-chloro-biphenyl, ammonium salt	C12H11ClFNO4S	FC1=C(C=CC(C2=CC=C(C=C2)OS(=O)([O-])=O)=C1)Cl.[NH4+]	InChI=1S/C12H8ClFO4S.H3N/c13-11-6-3-9(7-12(11)14)8-1-4-10(5-2-8)18-19(15,16)17;/h1-7H,(H,15,16,17);1H3	MUYMCEBMJRPJFT-UHFFFAOYSA-N	NA	NA	NA	NA	NA	NA
PCB30	2,4,6-Trichlorobiphenyl	C12H7Cl3	C1=CC=C(C=C1)C2=C(C=C(C=C2Cl)Cl)Cl	InChI=1S/C12H7Cl3/c13-9-6-10(14)12(11(15)7-9)8-4-2-1-3-5-8/h1-7H	MTLMVEWEYZFYTH-UHFFFAOYSA-N	35693-92-6	https://commonchemistry.cas.org/detail?cas_rn=35693-92-6	37247	https://pubchem.ncbi.nlm.nih.gov/compound/37247	DTXSID7073482	https://comptox.epa.gov/dashboard/chemical/details/DTXSID7073482
PCB52	2,2',5,5'-Tetrachlorobiphenyl	C12H6Cl4	C1=CC(=C(C=C1Cl)C2=C(C=CC(=C2)Cl)Cl)Cl	InChI=1S/C12H6Cl4/c13-7-1-3-11(15)9(5-7)10-6-8(14)2-4-12(10)16/h1-6H	HCWZEPKLWVAEOV-UHFFFAOYSA-N	35693-99-3	https://commonchemistry.cas.org/detail?cas_rn=35693-99-3	37248	https://pubchem.ncbi.nlm.nih.gov/compound/37248	DTXSID3038305	https://comptox.epa.gov/dashboard/chemical/details/DTXSID3038305
4-OH-PCB52	2,5-dichloro-4-(2,5-dichlorophenyl)phenol	C12H6Cl4O	C1=CC(=C(C=C1Cl)C2=CC(=C(C=C2Cl)O)Cl)Cl	InChI=1S/C12H6Cl4O/c13-6-1-2-9(14)7(3-6)8-4-11(16)12(17)5-10(8)15/h1-5,17H	ZKDSNFDCQYBBIU-UHFFFAOYSA-N	51274-68-1	https://commonchemistry.cas.org/detail?cas_rn=51274-68-1	39971	https://pubchem.ncbi.nlm.nih.gov/compound/39971	DTXSID10199272	https://comptox.epa.gov/dashboard/DTXSID10199272
PCB77	3,3',4,4'-Tetrachlorobiphenyl	C12H6Cl4	C1=CC(=C(C=C1C2=CC(=C(C=C2)Cl)Cl)Cl)Cl	InChI=1S/C12H6Cl4/c13-9-3-1-7(5-11(9)15)8-2-4-10(14)12(16)6-8/h1-6H	UQMGJOKDKOLIDP-UHFFFAOYSA-N	32598-13-3	https://commonchemistry.cas.org/detail?cas_rn=32598-13-3	36187	https://pubchem.ncbi.nlm.nih.gov/compound/36187	DTXSID5022514	https://comptox.epa.gov/dashboard/chemical/details/DTXSID5022514
4'-OH-PCB159	2,6-dichloro-4-(2,3,4,5-tetrachlorophenyl)phenol	C12H4Cl6O	C1=C(C=C(C(=C1Cl)O)Cl)C2=CC(=C(C(=C2Cl)Cl)Cl)Cl	InChI=1S/C12H4Cl6O/c13-6-3-5(9(16)11(18)10(6)17)4-1-7(14)12(19)8(15)2-4/h1-3,19H	PZAKBNHYWBSZAF-UHFFFAOYSA-N	158076-63-2	https://chem.nlm.nih.gov/chemidplus/sid/0158076632	178005	https://pubchem.ncbi.nlm.nih.gov/compound/178005	DTXSID70166369	https://comptox.epa.gov/dashboard/DTXSID70166369
PCB204	2,2',3,4,4',5,6,6'-Octachlorobiphenyl	C12H2Cl8	C1=C(C=C(C(=C1Cl)C2=C(C(=C(C(=C2Cl)Cl)Cl)Cl)Cl)Cl)Cl	InChI=1S/C12H2Cl8/c13-3-1-4(14)6(5(15)2-3)7-8(16)10(18)12(20)11(19)9(7)17/h1-2H	JDZUWXRNKHXZFE-UHFFFAOYSA-N	74472-52-9	https://commonchemistry.cas.org/detail?cas_rn=74472-52-9	91721	https://pubchem.ncbi.nlm.nih.gov/compound/91721	DTXSID7074240	https://comptox.epa.gov/dashboard/chemical/details/DTXSID7074240

Table S2. Summary statistics of body and organ weights (n= 6 per sex and exposure). Data in grams are reported as mean ± standard deviation.

Exposure Group	Sex	Body Weight Post Exposure	Liver	Lung	Thymus	Spleen	Kidneys	Adrenal Glands	Reproductive
Sham	Male	241.78 ± 6.94	11.03 ± 0.76	1.36 ± 0.13	0.55 ± 0.07	0.60 ± 0.05	2.29 ± 0.17	0.05 ± 0.01	2.35 ± 0.31
	Female	196.57 ± 11.46	8.27 ± 0.52	1.17 ± 0.12	0.60 ± 0.08	0.48 ± 0.06	1.94 ± 0.11	0.07 ± 0.01	0.63 ± 0.21
Low	Male	228.20 ± 14.09	10.31 ± 0.74	1.48 ± 0.29	0.70 ± 0.15	0.59 ± 0.04	1.99 ± 0.18	0.04 ± 0.01	2.51 ± 0.14
	Female	183.46 ± 14.31	8.11 ± 0.60	1.24 ± 0.13	0.61 ± 0.12	0.47 ± 0.10	1.70 ± 0.31	0.04 ± 0.01	0.55 ± 0.10
High	Male	192.17 ± 22.01	9.28 ± 1.41	1.52 ± 0.47	0.56 ± 0.05	0.53 ± 0.10	1.73 ± 0.23	0.03 ± 0.01	1.82 ± 0.52
	Female	196.92 ± 9.39	8.29 ± 0.72	1.23 ± 0.22	0.58 ± 0.06	0.44 ± 0.05	1.85 ± 0.25	0.06 ± 0.01	0.59 ± 0.16

Table S3. Summary of organ weights adjusted for body weight (n= 6 per sex and exposure). Data are reported as mean (standard deviation).

**Relative Organ Weights (g/g BW)**	**Males**			**Females**		
	**Sham**	**Low**	**High**	**Sham**	**Low**	**High**
Brain	0.008 (0.001)	0.008 (0.001)	0.010 (0.001)	0.010 (0.001)	0.010 (0.001)	0.010 (0.001)
Liver	0.046 (0.002)	0.045 (0.002)	0.048 (0.003)	0.042 (0.003)	0.044 (0.002)	0.042 (0.003)
Lung	0.006 (0.001)	0.007 (0.002)	0.008 (0.002)	0.006 (0.001)	0.007 (0.001)	0.006 (0.001)
Thymus	0.002 (0.001)	0.003 (0.001)	0.003 (0.001)	0.003 (0.001)	0.003 (0.001)	0.003 (0.001)
Spleen	0.003 (0.001)	0.003 (0.001)	0.003 (0.001)	0.002 (0.001)	0.003 (0.001)	0.002 (0.001)
Kidneys	0.009 (0.001)	0.009 (0.001)	0.009 (0.001)	0.010 (0.001)	0.009 (0.001)	0.009 (0.001)
Testies	0.010 (0.001)	0.011 (0.001)	0.009 (0.002)	-	-	-
Ovaries	-	-	-	0.004 (0.001)	0.003 (0.001)	0.003 (0.001)

BW, body weight

Table S4. Recoveries (%) of the Ongoing Precision and Recovery standard spiked into matrices from naïve animals in the GC-MS/MS analysis. Data are reported as mean (standard deviation).

PCB or Metabolite	Blank Spike							Tissue Blank Spike						
	**Adipose**, n= 4	**Brain**, n= 6	**Cecum**, n= 3	**Intestinal Content**, n= 9	**Liver**, n= 9	**Lung**, n= 5	**Serum**, n= 6	**Adipose**, n= 4	**Brain**, n= 6	**Cecum**, n= 3	**Intestinal Content**, n= 9	**Liver**, n= 9	**Lung**, n= 5	**Serum**, n= 6
PCB52	85 (27)	99 (18)	188 (8)	76 (21)	86 (27)	91 (17)	80 (21)	98 (31)	94 (13)	166 (21)	97 (23)	92 (30)	100 (12)	85 (23)
4-OH-PCB52	78 (11)	83 (12)	107 (5)	94 (23)	77 (9)	81 (8)	83 (22)	86 (16)	75 (12)	115 (18)	108 (16)	87 (12)	84 (6)	75 (17)
Mean (SD)														

N represents the number of samples analyzed. Recoveries are a culmination of three pools of data. Adipose, brain, liver, and serum data has been previously reported[13] and published at DOI: 10.25820/data.006225.

Table S5. Method detection limits (ng) and limits of detection for each tissue type (ng/g) for the gas chromatographic quantifications of PCB52 and 4-OH-PCB52.

Tissue LODs	PCB52	4-OH-PCB52	X1-OH-PCB52
**MDL [ng][1]**	0.08	0.04	0.03
**Adipose**			
LOD[2] [ng]	0.04	0.01	0.01
LOD[3] [ng/g]	0.4	0.1	0.1
**Brain**			
LOD[2] [ng]	0.2	0.01	0.02
LOD[3] [ng/g]	0.2	0.01	0.02
**Cecum**			
LOD[2] [ng]	0.1	0.05	1.3
LOD[3] [ng/g]	0.2	0.1	2.5
**Intestinal content**			
LOD[2] [ng]	0.04	0.01	0.01
LOD[3] [ng/g]	0.8	0.2	0.1
**Liver**			
LOD[2] [ng]	0.05	0.02	0.03
LOD[3] [ng/g]	0.09	0.03	0.06
**Lung**			
LOD[2] [ng]	0.06	0.02	0.02
LOD[3] [ng/g]	0.1	0.04	0.04
**Serum**			
LOD[2] [ng]	0.1	0.03	0.06
LOD[3] [ng/g]	0.3	0.05	0.1

[1] MDL, method detection limit, was calculated based on method blanks.

[2] LOD, limit of detection, was calculated based on matrix blanks for each tissue using the formula LOD= $mean_{blank} + t_{0.01,n-1=8} * SD_{blank}$, where $mean_{blank}$ is the mean of nine blank measures, $t_{0.01,n-1=8}$ is Student's t-value for n – 1 degrees of freedom at the 99% confidence level, and SD_{blank} is the standard deviation of the blank measures.

[3] LOD adjusted for tissue weight (g).

Table S6. Surrogate standard recoveries (%) from all tissues in the GC-MS/MS analysis.

PCB or Metabolite		Low dose							High dose						
		Adipose	Brain	Cecum	Intestinal content	Liver	Lung	Serum	Adipose	Brain	Cecum	Intestinal content	Liver	Lung	Serum
PCB52	Male	6 (2)	1 (0.4)	1 (1)	1 (NA)	9 (3)	50 (9)	2 (1)	8 (5)	2 (1)	2 (3)	1 (0.1)	10 (2)	46 (23)	2 (1)
	Female	6 (3)	2 (1)	1 (1)	5 (NA)	7 (3)	41 (12)	1 (0.3)	6 (5)	2 (0)	1 (1)	2 (1)	7 (2)	45 (11)	1 (0.3)
4-OH-PCB52	Male	NA (NA)	NA (NA)	1 (1)	NA (NA)	0.1 (0.1)	0.1 (0.03)	0.4 (0.1)	0.2 (0.01)	NA (NA)	2 (2)	0.4 (0.1)	0.3 (0.2)	0.2 (0.1)	0.4 (0.2)
	Female	NA (NA)	NA (NA)	2 (1)	4 (6)	0.2 (0.1)	0.1 (0.04)	0.4 (0.1)	0.2 (NA)	NA (NA)	3 (3)	5 (8)	0.2 (0.05)	0.2 (0.06)	0.1 (0.1)

Recovery Standard	Adipose, n= 24	Brain, n= 24	Cecum, n= 24	Intestinal content, n= 24	Liver, n= 24	Lung, n= 24	Serum, n= 24
**PCB77**							
Mean (SD)	96 (7)	101 (22)	109 (15)	103 (14)	85 (11)	84 (8)	103 (24)
Range	82, 112	59, 159	90, 133	77, 144	41, 95	68, 94	66, 147
**4'-OH-PCB159**							
Mean (SD)	95 (5)	91 (11)	95 (6)	92 (11)	87 (5)	88 (3)	80 (17)
Range	82, 106	70, 114	82, 109	59, 104	75, 94	83, 95	58, 120
**3-F-PCB3 sulfate**							
Mean (SD)			64 (40)	52 (14)			
Range			10, 151	1, 73			

PCB77, a tetra chlorinated biphenyl, was used as a surrogate standard for PCB52. 4'-OH-PCB159 was used as a surrogate standard for hydroxylated PCB metabolites to determine extraction efficiency. 3-F-PCB3 sulfate was a surrogate standard to assess the recovery of PCB sulfates from deconjugation experiments.

X1-OH-PCB52	Male	NA (NA)	NA (NA)	15 (10)	1 (1)	1 (0.2)	1 (0.3)	0.3 (0.1)	NA (NA)	NA (NA)	20 (20)	3 (2)	1 (0.4)	1 (0.2)	0.2 (0.04)
	Female	0 (NA)	NA (NA)	26 (17)	22 (43)	1 (0.2)	1 (0.3)	0.2 (0.01)	0.2 (0.1)	NA (NA)	34 (34)	28 (60)	1 (0.2)	1 (0.3)	0.2 (0.04)

Table S7. PCB or metabolite ng/g wet weight detected levels of each compound across tissue and exposure group (n= 6 per sex and

X1-OH-PCB52, unidentified monohydroxylated PCB52 metabolite.
NA, not available either because detection frequency was 0 for mean or 1 for standard deviation.
exposure). Data are reported as mean (standard deviation).

Table S8. Precursor ions, product ions, and collision energies for each analyte included in the GC/MS/MS analysis.

**Analyte**	**Precursor Ion (*m/z*)**	**Product Ion (*m/z*)**	**Collision Energy (eV)**
PCB52	291.9	222.0	25
4-OH-PCB52	321.9	278.9	20
PCB77	291.9	222.0	25
PCB204	429.7	357.8	35
4'-OH-PCB159	389.9	374.9	15

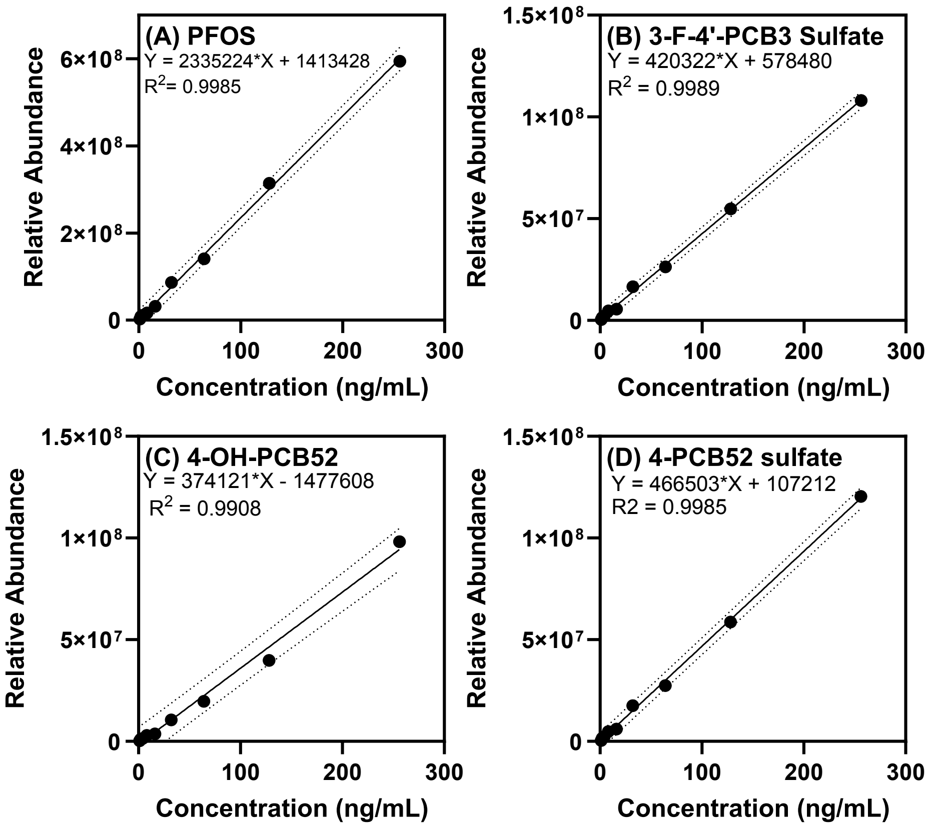

Figure S1. Calibration curve of (A) PFOS, (B) 3-F-4'-PCB3 sulfate, (C) 4-OH-PCB52 and (D) 4-PCB52 sulfate showed that these compounds are linear in the range of 1 to 256 ng/mL in acetonitrile-H_2O, 1:1 (v/v). The relative abundance values are the peak area of each analyte. A simple linear regression analyses were performed with GraphPad prism 10.0.2.

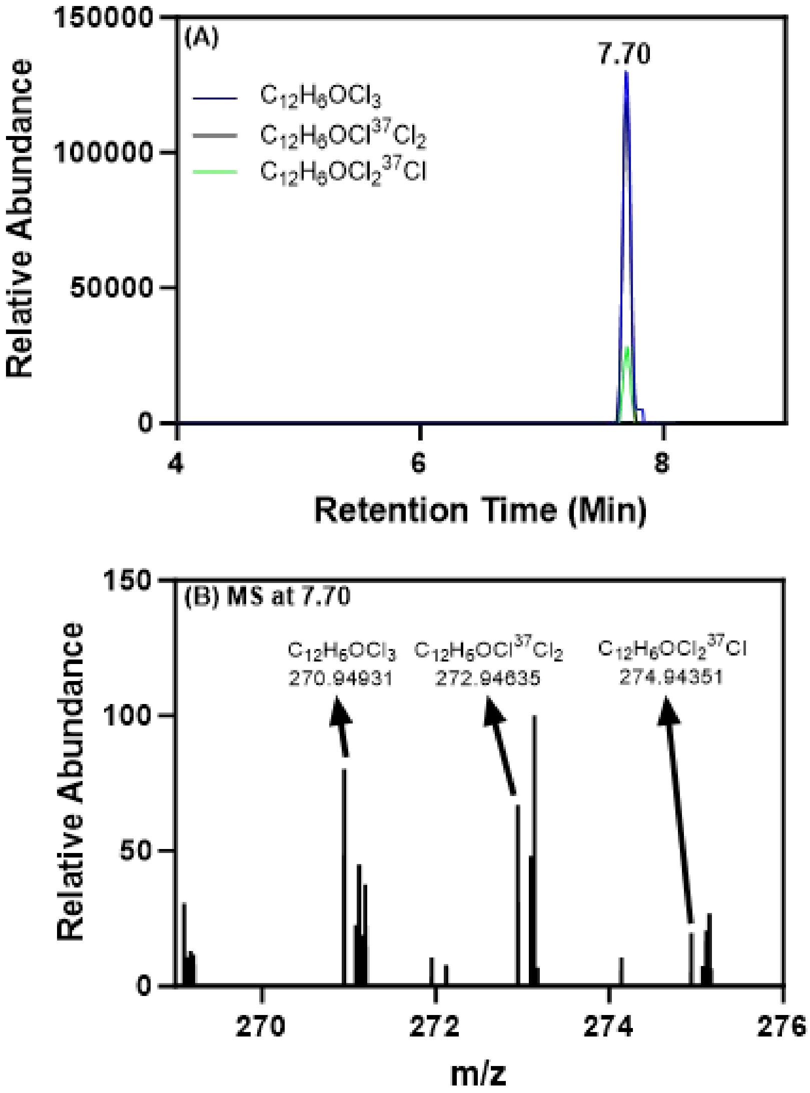

Figure S2. One trichlorinated OH-PCB metabolite was detected by LC-Orbitrap MS in the intestinal content of PCB52-exposed rats. (A) Chromatograms extracted based on the theoretical accurate mass of the top three high-abundance isotope ions of trichlorinated OH-PCBs ($[C_{12}H_7OCl_3]^-$, *m/z* 270.94897 for the monoisotopic ion) show a peak at 7.70 min. (B) The accurate masses of three high-abundance isotope ions at 7.70 min match the theoretical accurate mass and isotopic pattern (3:3:1) of a trichlorinated compound. The LC-Orbitrap MS analysis was performed in the negative polarity mode.

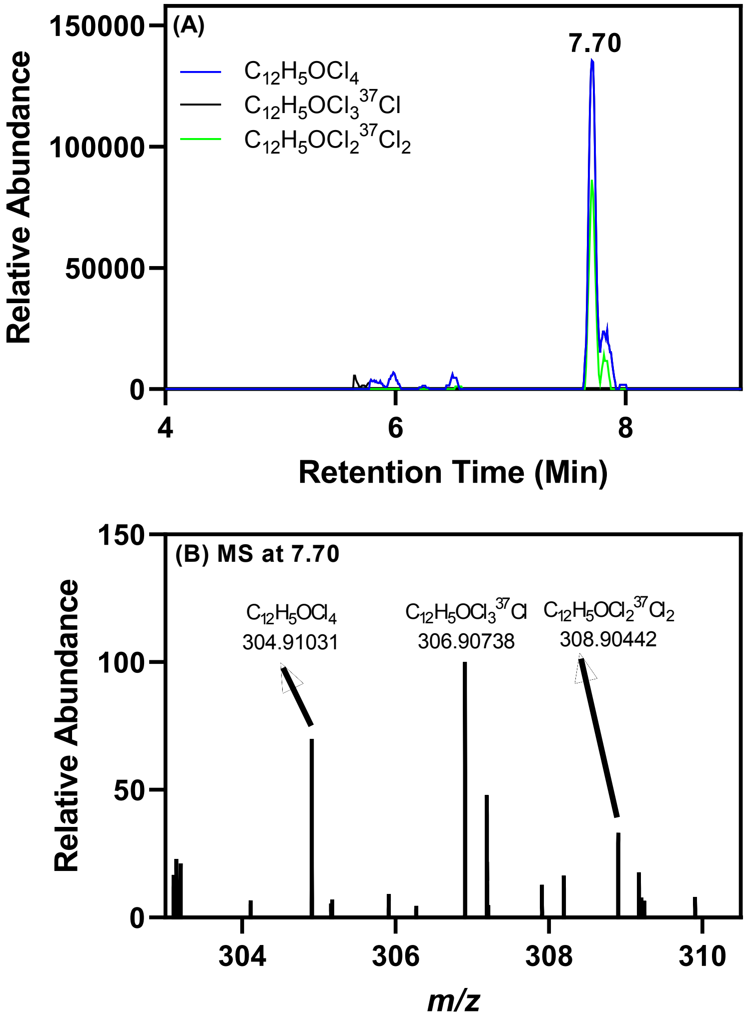

Figure S3. One tetrachlorinated OH-PCB metabolite was detected by LC-Orbitrap MS in the intestinal content of PCB52-exposed rats. (A) Chromatograms extracted based on the theoretical accurate mass of the top three high-abundance isotope ions of tetrachlorinated OH-PCBs ($[C_{12}H_5OCl_4]^-$, *m/z* 304.91000 for the monoisotopic ion) show a peak at 7.70 min. (B) The accurate masses of three high-abundance isotope ions at 7.70 min match the theoretical accurate mass and isotopic pattern (8:10:5) of a tetrachlorinated compound. The LC-Orbitrap MS analysis was performed in the negative polarity mode.

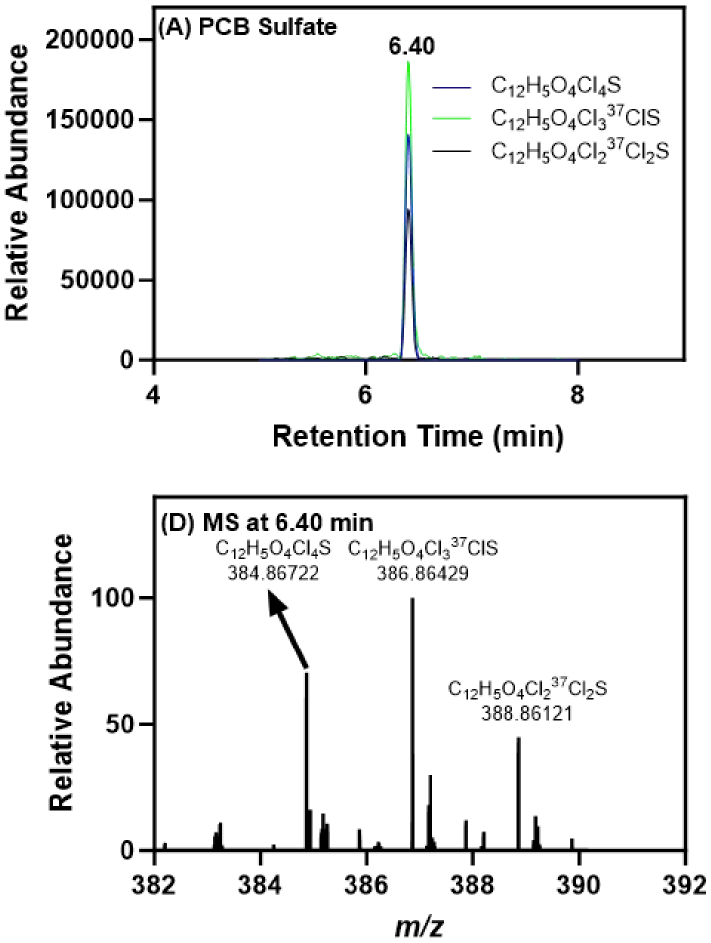

Figure S4. One tetrachlorinated PCB sulfate was detected by LC-Orbitrap MS in the lung of PCB52-exposed rats. (A) Chromatograms extracted based on the theoretical accurate mass of the top three high-abundance isotope ions of tetrachlorinated PCB sulfates ($[C_{12}H_5Cl_4O_4S]^-$, *m/z* 384.86681 for the monoisotopic ion) show a peak at 6.40 min. The accurate masses of three high-abundance isotope ions at (B) 6.40 min match the theoretical accurate mass and isotopic pattern (8:10:5) of a tetrachlorinated compound. The LC-Orbitrap MS analysis was performed in the negative polarity mode.

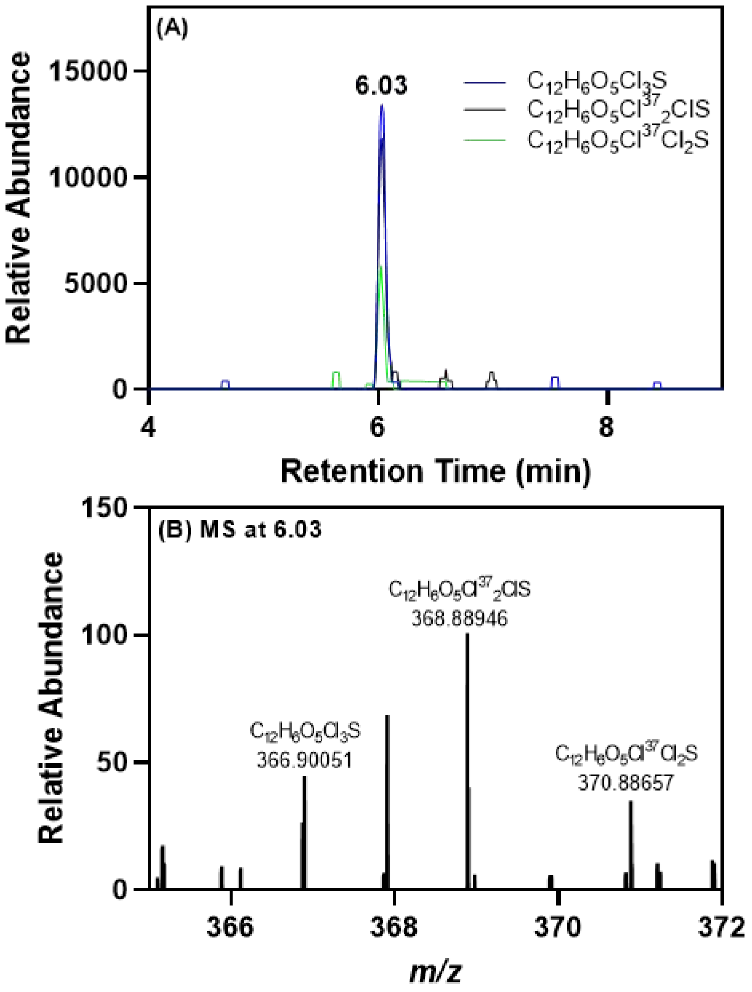

Figure S5. One trichlorinated OH-PCB sulfate was detected by LC-Orbitrap MS in the serum of PCB52-exposed rats. (A) Chromatograms extracted based on the theoretical accurate mass of the top three high-abundance isotope ions of trichlorinated OH-PCB sulfates ($[C_{12}H_6O_5Cl_3S]^-$, *m/z* 366.90070for the monoisotopic ion) show a peak at 6.03 min. The accurate masses of three high-abundance isotope ions at (B) 6.03 min match the theoretical accurate mass and isotopic pattern (3:3:1) of a trichlorinated compound. The LC-Orbitrap MS analysis was performed in the negative polarity mode.

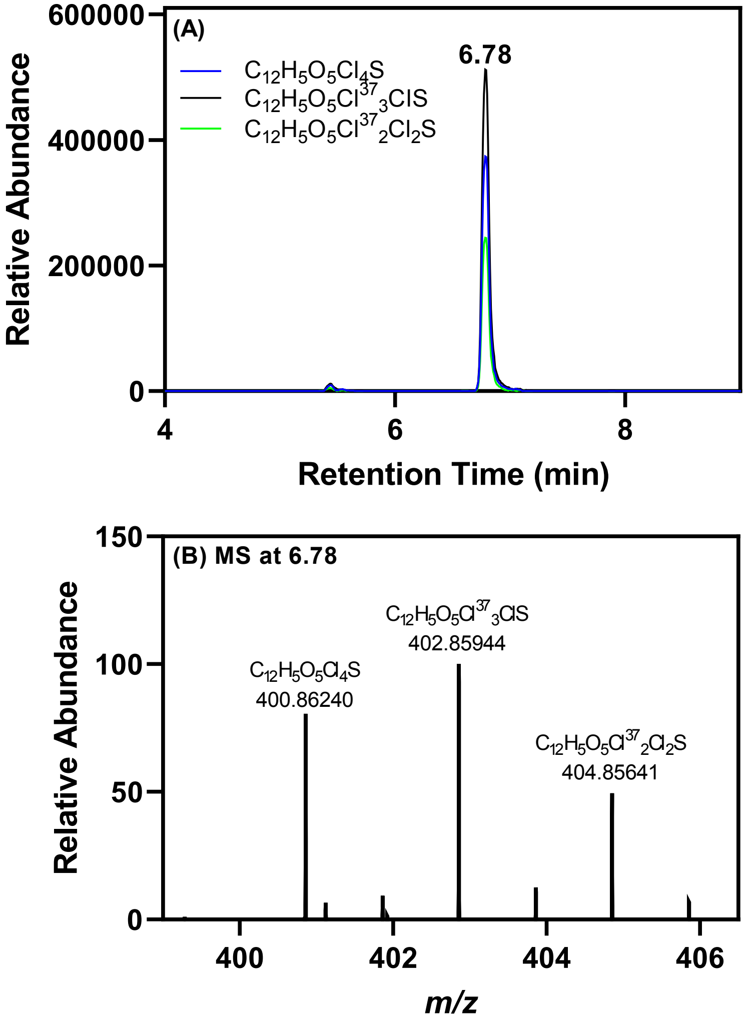

Figure S6. One tetrachlorinated OH-PCB sulfates was detected by LC-Orbitrap MS in the liver of PCB52-exposed rats. (A) Chromatograms extracted based on the theoretical accurate mass of the top three high-abundance isotope ions of tetrachlorinated OH-PCB sulfate ($[C_{12}H_5O_5Cl_4S]^-$, *m/z* 400.86173 for the monoisotopic ion) show a peak at 6.78 min. The accurate masses of several high-abundance isotope ions at (B) 6.78 min match the theoretical accurate mass and isotopic pattern (8:10:5) of a trichlorinated compound. The LC-Orbitrap MS analysis was performed in the negative polarity mode.

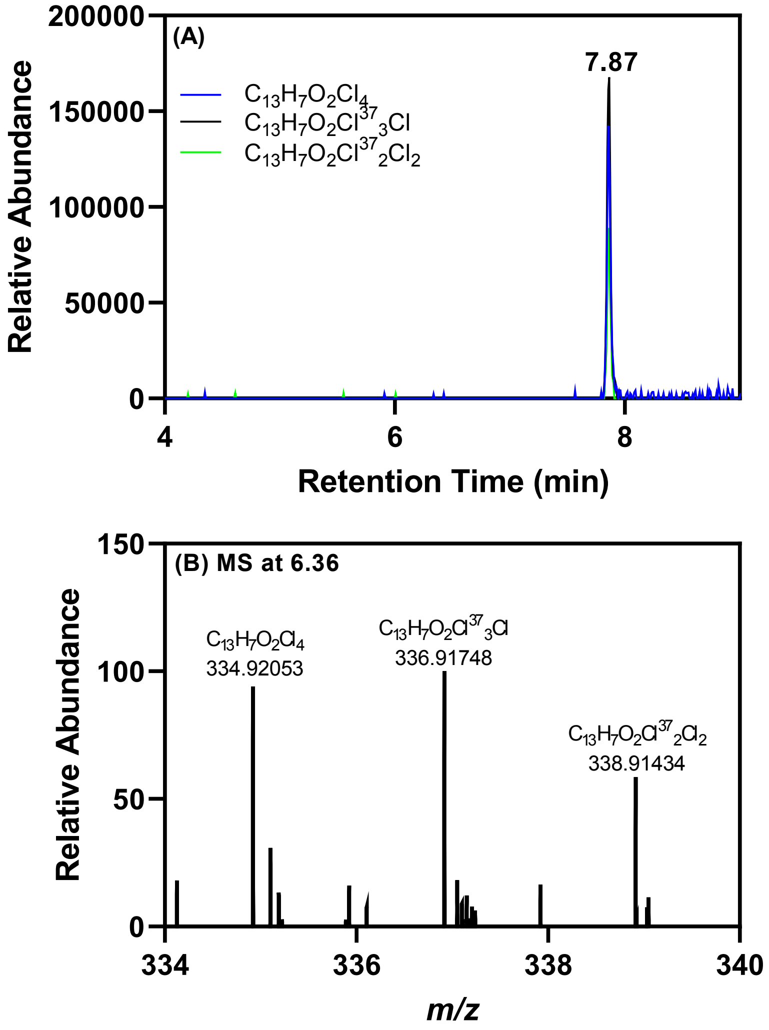

Figure S7. One tetrachlorinated MeO-OH-PCB was detected by LC-Orbitrap MS in the serum of PCB52-exposed rats. (A) Chromatograms extracted based on the theoretical accurate mass of the top three high-abundance isotope ions of tetrachlorinated MeO-OH-PCBs ($[C_{13}H_7O_3Cl_4]^-$, *m/z* 334.92056 for the monoisotopic ion) show a peak at 7.87 min. (B) The accurate masses of three high-abundance isotope ions at 7.87 min match the theoretical accurate mass and isotopic pattern (3:3:1) of a tetrachlorinated compound. The LC-Orbitrap MS analysis was performed in the negative polarity mode.

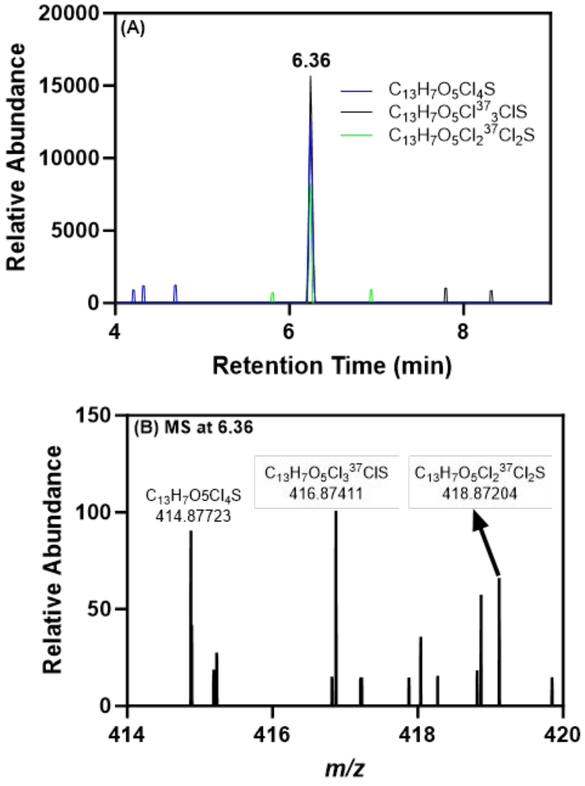

Figure S8. One tetrachlorinated MeO-PCB sulfate was detected by LC-Orbitrap MS in the serum of PCB52-exposed rats. (A) Chromatograms extracted based on the theoretical accurate mass of the top three high-abundance isotope ions of tetrachlorinated MeO-OH-PCB sulfates ($[C_{13}H_7O_5Cl_4S]^-$, *m/z* 414.87738for the monoisotopic ion) show a peak at 6.36 min. (B) The accurate masses of several high-abundance isotope ions at 6.36 min match the theoretical accurate mass and isotopic pattern (8:10:5) of a tetrachlorinated compound. The LC-Orbitrap MS analysis was performed in the negative polarity mode.

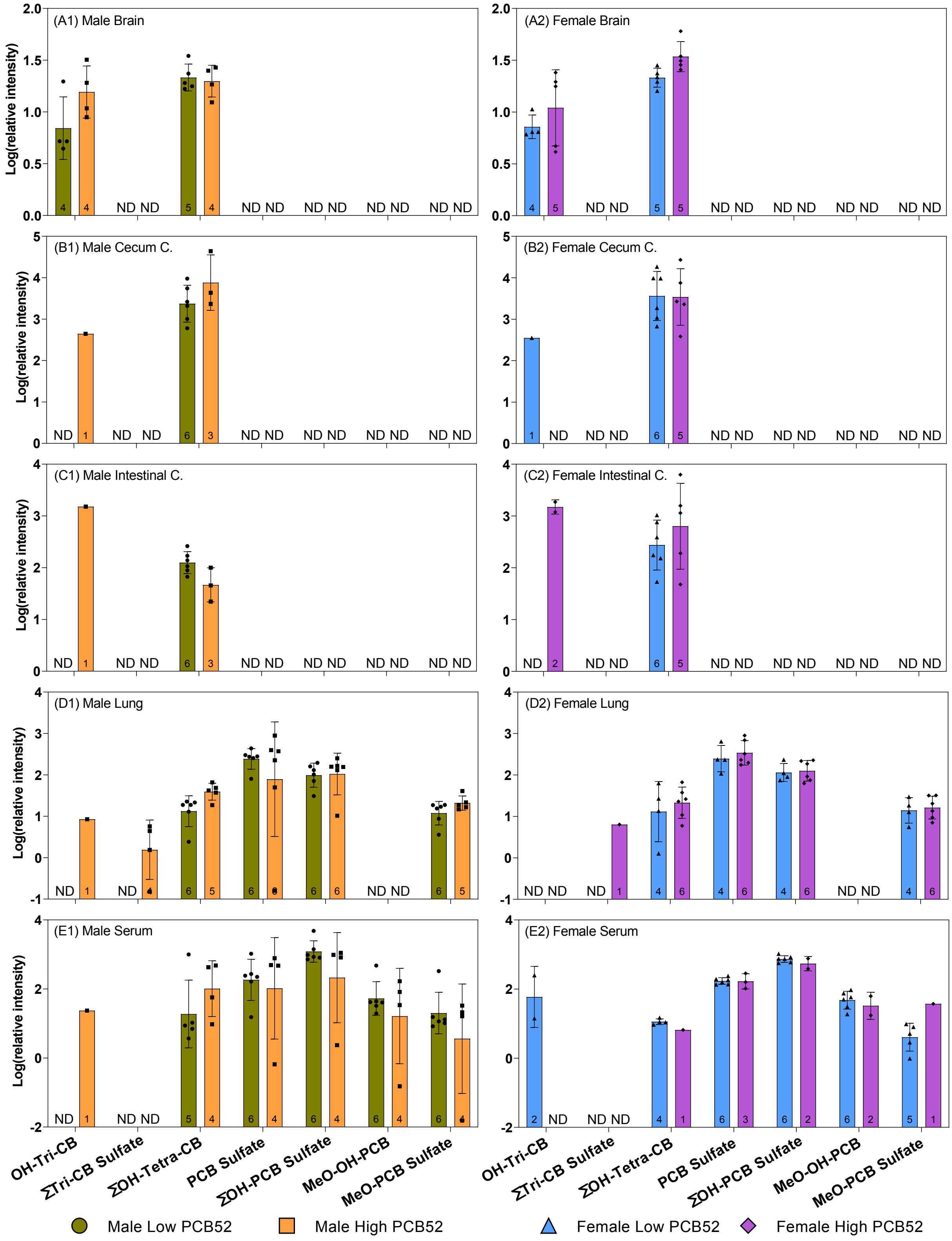
(A1) Male Brain
(A2) Female Brain
(B1) Male Cecum C.
(B2) Female Cecum C.
(C1) Male Intestinal C.
(C2) Female Intestinal C.
(D1) Male Lung
(D2) Female Lung
(E1) Male Serum
(E2) Female Serum
Log(relative intensity)
ND
OH-Tri-CB
ΣTri-CB Sulfate
ΣOH-Tetra-CB
PCB Sulfate
ΣOH-PCB Sulfate
MeO-OH-PCB
MeO-PCB Sulfate
Male Low PCB52
Male High PCB52
Female Low PCB52
Female High PCB52

Figure S9. The relative levels of PCB52 metabolites from LC-HRMS show distinct differences by compartment but not sex in rats exposed for 4 h to PCB52. (A1) male and (A2) female brain, (B1) male and (B2) female cecum content, (C1) male and (C2) female intestinal content, (D1) male and (D2) female lung, and (E1) male and (E2) female serum from rats exposed for 4 h by inhalation to PCB52. Data are expressed as the relative peak area adjusted for the peak area of PFOS and the tissue wet weight and represented as mean ± standard deviation on a logarithmic scale. The sum of several metabolites was plotted to simplify the metabolite profiles. Logistic regression was employed to investigate potential sex and/or dose effects within tissues. ND, not detected.

References

(1) Li, X.; Holland, E. B.; Feng, W.; Zheng, J.; Dong, Y.; Pessah, I. N.; Duffel, M. W.; Robertson, L. W.; Lehmler, H.-J. Authentication of synthetic environmental contaminants and their (bio)transformation products in toxicology: Polychlorinated biphenyls as an example. *Environ Sci Pollut Res Int* **2018**, *25* (17), 16508-16521.

(2) Saktrakulkla, P.; Li, X.; Martinez, A.; Lehmler, H.-J.; Hornbuckle, K. C. Hydroxylated polychlorinated biphenyls are emerging legacy pollutants in contaminated sediments. *Environ Sci Technol* **2022**, *56* (4), 2269-2278.

(3) Sethi, S.; Morgan, R. K.; Feng, W.; Lin, Y.; Li, X.; Luna, C.; Koch, M.; Bansal, R.; Duffel, M. W.; Puschner, B.; et al. Comparative analyses of the 12 most abundant PCB congeners detected in human maternal serum for activity at the thyroid hormone receptor and ryanodine receptor. *Environ Sci Technol* **2019**, *53* (7), 3948-3958.

(4) Rodriguez, E. A.; Li, X.; Lehmler, H. J.; Robertson, L. W.; Duffel, M. W. Sulfation of lower chlorinated polychlorinated biphenyls increases their affinity for the major drug-binding sites of human serum albumin. *Environ Sci Technol* **2016**, *50* (10), 5320-5327.

(5) Shaikh, N. S.; Parkin, S.; Luthe, G.; Lehmler, H. J. The three-dimensional structure of 3,3',4,4'-tetrachlorobiphenyl, a dioxin-like polychlorinated biphenyl (PCB). *Chemosphere* **2008**, *70* (9), 1694-1698.

(6) Tampal, N.; Lehmler, H. J.; Espandiari, P.; Malmberg, T.; Robertson, L. W. Glucuronidation of hydroxylated polychlorinated biphenyls (PCBs). *Chem Res Toxicol* **2002**, *15* (10), 1259-1266.

(7) Dhakal, K.; He, X.; Lehmler, H.-J.; Teesch, L. M.; Duffel, M. W.; Robertson, L. W. Identification of sulfated metabolites of 4-chlorobiphenyl (PCB3) in the serum and urine of male rats. *Chem Res Toxicol* **2012**, *25* (12), 2796-2804.

(8) Li, X.; Hefti, M. M.; Marek, R. F.; Hornbuckle, K. C.; Wang, K.; Lehmler, H.-J. Assessment of polychlorinated biphenyls and their hydroxylated metabolites in postmortem human brain samples: Age and brain region differences. *Environ Sci Technol* **2022**, *56* (13), 9515-9526.

(9) Kania-Korwel, I.; Zhao, H.; Norstrom, K.; Li, X.; Hornbuckle, K. C.; Lehmler, H. J. Simultaneous extraction and clean-up of polychlorinated biphenyls and their metabolites from small tissue samples using pressurized liquid extraction. *J Chromatogr A* **2008**, *1214* (1-2), 37-46.

(10) Wu, X.; Barnhart, C.; Lein, P. J.; Lehmler, H. J. Hepatic metabolism affects the atropselective disposition of 2,2',3,3',6,6'-hexachlorobiphenyl (PCB 136) in mice. *Environ Sci Technol* **2015**, *49* (1), 616-625.

(11) Wang, H.; Adamcakova-Dodd, A.; Flor, S.; Gosse, L.; Klenov, V. E.; Stolwijk, J. M.; Lehmler, H. J.; Hornbuckle, K. C.; Ludewig, G.; Robertson, L. W.; et al. Comprehensive subchronic inhalation toxicity assessment of an indoor school air mixture of PCBs. *Environ Sci Technol* **2020**, *54* (24), 15976-15985.

(12) Wang, H.; Adamcakova-Dodd, A.; Lehmler, H. J.; Hornbuckle, K. C.; Thorne, P. S. Toxicity assessment of 91-day repeated inhalation exposure to an indoor school air mixture of PCBs. *Environ Sci Technol* **2022**, *56* (3), 1780-1790.

(13) Bullert, A.; Li, X.; Chunyun, Z.; Lee, K.; Pulliam, C. F.; Cagle, B. S.; Doorn, J. A.; Klingelhutz, A. J.; Robertson, L. W.; Lehmler, H.-J. Disposition and metabolomic effects

of 2,2′,5,5′-tetrachlorobiphenyl in female rats following intraperitoneal exposure. *Environ Toxicol Pharmacol* **2023**, *102*, 104245.
